# Glucose-dependent insulinotropic polypeptide receptor signaling alleviates gut inflammation in mice

**DOI:** 10.1172/jci.insight.174825

**Published:** 2024-12-26

**Authors:** Rola Hammoud, Kiran Deep Kaur, Jacqueline A. Koehler, Laurie L. Baggio, Chi Kin Wong, Katie E. Advani, Bernardo Yusta, Irina Efimova, Fiona M. Gribble, Frank Reimann, Sigal Fishman, Chen Varol, Daniel J. Drucker

**Affiliations:** 1 Lunenfeld-Tanenbaum Research Institute, Sinai Health System, University of Toronto, Toronto, Ontario, Canada.; 2 The Research Center for Digestive Tract and Liver Diseases, Tel-Aviv Sourasky Medical Center, Tel Aviv, Israel.; 3 Department of Clinical Microbiology and Immunology, Faculty of Medical and Health Sciences, Tel-Aviv University, Tel-Aviv, Israel.; 4 Metabolic Research Laboratories, Institute of Metabolic Science, University of Cambridge, Addenbrooke’s Hospital, Hills Road, Cambridge, United Kingdom.

**Keywords:** Endocrinology, Diabetes

## Abstract

Glucose-dependent insulinotropic polypeptide (GIP) and glucagon-like peptide 1 (GLP-1) are gut-derived peptide hormones that potentiate glucose-dependent insulin secretion. The clinical development of GIP receptor–GLP-1 receptor (GIPR–GLP-1R) multiagonists exemplified by tirzepatide and emerging GIPR antagonist–GLP-1R agonist therapeutics such as maritide is increasing interest in the extrapancreatic actions of incretin therapies. Both GLP-1 and GIP modulate inflammation, with GLP-1 also acting locally to alleviate gut inflammation in part through antiinflammatory actions on GLP-1R^+^ intestinal intraepithelial lymphocytes. In contrast, whether GIP modulates gut inflammation is not known. Here, using gain- and loss-of-function studies, we show that GIP alleviates 5-fluorouracil–induced (5FU-induced) gut inflammation, whereas genetic deletion of *Gipr* exacerbates the proinflammatory response to 5FU in the murine small bowel (SB). Bone marrow (BM) transplant studies demonstrated that BM-derived *Gipr*-expressing cells suppress 5FU-induced gut inflammation in the context of global *Gipr* deficiency. Within the gut, *Gipr* was localized to nonimmune cells, specifically stromal CD146^+^ cells. Hence, the extrapancreatic actions of GIPR signaling extend to the attenuation of gut inflammation, findings with potential translational relevance for clinical strategies modulating GIPR action in people with type 2 diabetes or obesity.

## Abstract 2

Here we demonstrate that activation vs. loss of GIP action impacts the extent of experimental gut inflammation in mice

## Graphical abstract

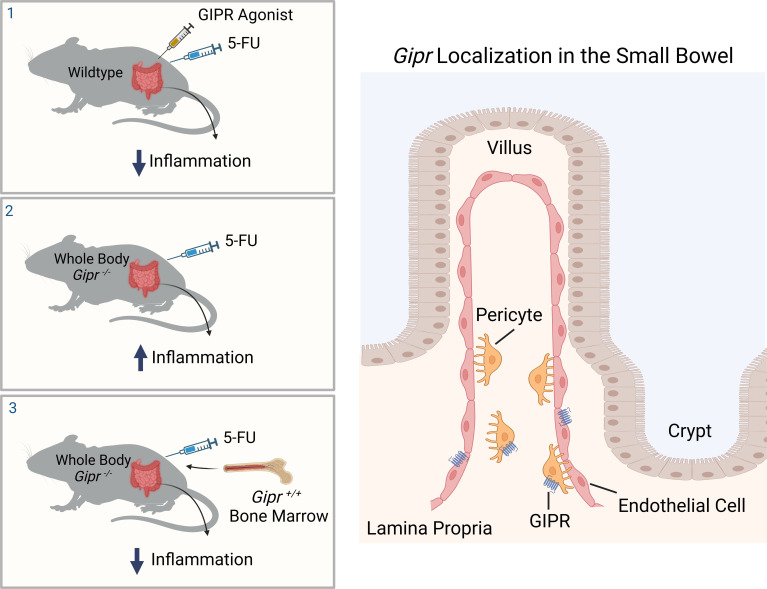

## Introduction

Glucose-dependent insulinotropic polypeptide (GIP) and glucagon-like peptide 1 (GLP-1) are incretin hormones secreted from enteroendocrine K and L cells, respectively, that potentiate insulin secretion from the pancreas (1). GLP-1 and GIP also act on the brain to reduce food intake and promote weight loss (2). GLP-1R agonists (GLP-1RA) are utilized clinically for the treatment of type 2 diabetes (T2D) and obesity (2, 3) and a single GIP receptor–GLP-1 receptor (GIPR–GLP-1R) coagonist, tirzepatide (TZP), is approved for the treatment of T2D (2, 4) and obesity (5).

GLP-1R agonism also reduces systemic and gut inflammation (6, 7), potentially contributing to reduction of the complications associated with metabolic diseases (5, 8). Preliminary clinical evidence suggests a role for GLP-1RA and dipeptidyl peptidase 4 (DPP-4) inhibitors in the reduction of adverse clinical events in patients with T2D diagnosed with inflammatory bowel disease (IBD), such as lower rates of IBD-related hospitalizations, IBD-related major surgery, and reduced reliance on oral corticosteroids and TNF-α inhibitor drugs (9). In contrast, much less is known about the actions of GIP to reduce inflammation in different tissue compartments. GIPR expression has been localized to myeloid cells derived from the bone marrow (BM) (10–12), and GIPR agonism reduces — whereas loss of GIPR action enhances — adipose tissue inflammation, in part through mechanisms involving BM-derived *Gipr*-expressing macrophages (11, 12).

Beyond their classical actions as incretin hormones, GIP and GLP-1 also exert actions in the gut. GLP-1 decreases gastrointestinal motility (13), reduces postprandial secretion of gastric acid and enterocyte-derived chylomicrons (14, 15), and alleviates experimental gut inflammation (7, 16). Conversely, loss of the GLP-1 receptor in *Glp1r*^–/–^ mice exacerbates the extent of mucosal gut injury and intestinal inflammation (16). Moreover, GLP-2 cosecreted with GLP-1 from gut L cells also exerts local antiinflammatory actions and improves gut barrier function to reduce both intestinal and systemic inflammation (17, 18). The actions of GIP in the gut are more limited and include reduction of gut motility and intestinal glucose absorption in preclinical studies (19). However, whether GIP also controls gut inflammation has not been determined.

Since GIP modulates macrophage-driven inflammation in adipose tissue through actions on BM-derived myeloid cells (11), we hypothesized that, like GLP-1 and GLP-2, GIP might also exert antiinflammatory actions in the gut. We previously studied the BM response to gain and loss of GIPR signaling in mice treated with 5-fluorouracil (5FU) (12), a widely used chemotherapeutic agent that disrupts DNA synthesis through the inhibition of thymidylate synthase, leading to reduced cellular replication and apoptosis, often associated with intestinal injury, diarrhea, and intestinal mucositis (20).

Here, we show that the GIPR agonist [D-Ala^2^]-GIP alleviates the proinflammatory response in a mouse model of 5FU-induced gut injury. Conversely, mice with whole-body deletion of the murine *Gipr* exhibit increased 5FU-induced gut inflammation, most prominently within the ileum. BM transplant studies reveal that mice with BM-specific *Gipr* deletion do not phenocopy the enhanced gut inflammation detected in 5FU-treated *Gipr^–/–^* mice. In contrast, BM-derived *Gipr*-expressing cells suppress inflammation in the context of global *Gipr* deficiency. *Gipr* expression is enriched in the lamina propria of the proximal, but not distal, small bowel (SB); however *Gipr* mRNA is not detected at higher levels in gut immune cells (i.e., CD45^+^ cells). Rather, we identify *Gipr* within CD146^+^ cells — i.e., pericytes and endothelial cells (ECs). These findings extend our understanding of the extrapancreatic actions of gain and loss of GIPR signaling to encompass the control of intestinal inflammation.

## Results

### Treatment with [D-Ala^2^]-GIP protects against 5FU-induced intestinal damage and inflammation.

**Figure 1:**
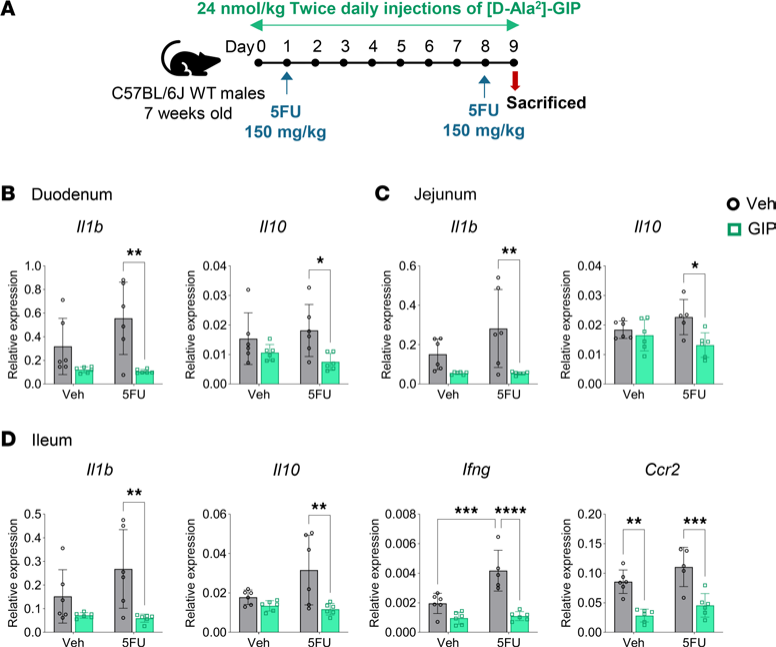
Treatment with [D-Ala^2^]-GIP downregulates cytokine gene expression in the small bowel of mice exposed to 5FU. (**A**) Schematic representation of the experimental protocol. (**B**–**D**) Gene expression, relative to *Tbp*, of cytokines in response to 5FU and [DAla^2^]-GIP coadministration within the duodenum, jejunum, and ileum (*n* = 5–6). Data are presented as mean ± SD of samples pooled from 3 independent mouse cohorts. *P ≤ 0.05, **P ≤ 0.01, ***P ≤ 0.001, and ****P ≤ 0.0001 by 2-way ANOVA followed by Tukey post hoc tests.

We previously determined that GIPR agonism regulates BM hematopoietic responses to 5FU and Pam3CysSerLys4 (Pam3CSK4), whereas loss of the *Gipr* dysregulated the hematopoietic response to 5FU but not to Pam3CSK4 or LPS (12). Analysis of the effect of these treatments on a subset of immunoregulatory gene expression profiles in the gut revealed that [D-Ala^2^]-GIP (hereafter referred to as GIP) did not modulate the immune response to LPS or Pam3CSK4 within the ileum and jejunum (data not shown). However, treatment with GIP downregulated cytokine gene expression in the SB of mice treated with a moderate dose of 5FU (150 mg/kg, injected twice, 1 week apart) (Figure 1, A–D), a dosing regimen originally selected to interrogate hematopoiesis (12). Levels of IL-1β (*Il1b*) and IL-10 (*Il10*) mRNA transcripts were reduced in the duodenum (Figure 1B) and jejunum (Figure 1C) of mice treated with GIP and 5FU; however, levels of TNF-α (*Tnf*), IFN-γ (*Ifng*), and chemokine receptor-2 (*Ccr2*) were not different (Supplemental Figure 1, A and B; supplemental material available online with this article; https://doi.org/10.1172/jci.insight.174825DS1). The immunoregulatory effects of GIP were most evident in the distal SB as *Il1b*, *Il10*, *Ifng*, and *Ccr2* mRNAs were downregulated by GIP in the ileum of 5FU-treated mice (Figure 1D). The ileal transcript level of *Tnf* (Supplemental Figure 1C) and the protein concentrations of IL-1β, IL-10, keratinocyte chemoattractant/human growth-regulated oncogene (KC/GRO), TNF-α, IL-6, and IFN-γ within the ileum and circulation were not different (Supplemental Figure 1, D and E). Mice cotreated with GIP and the moderate dose of 5FU had reduced body weight, but no differences in SB weight or the SB weight-to-length ratio (Supplemental Figure 2A). Furthermore, spleen weight of 5FU-treated mice was lower, irrespective of GIP treatment. Histology analysis showed a reduction in crypt depth in the ileum of vehicle-and GIP-treated mice exposed to 5FU, indicative of mild gut injury, though there were no differences between groups in villus height or crypt density (Supplemental Figure 2, B and C).

**Figure 2:**
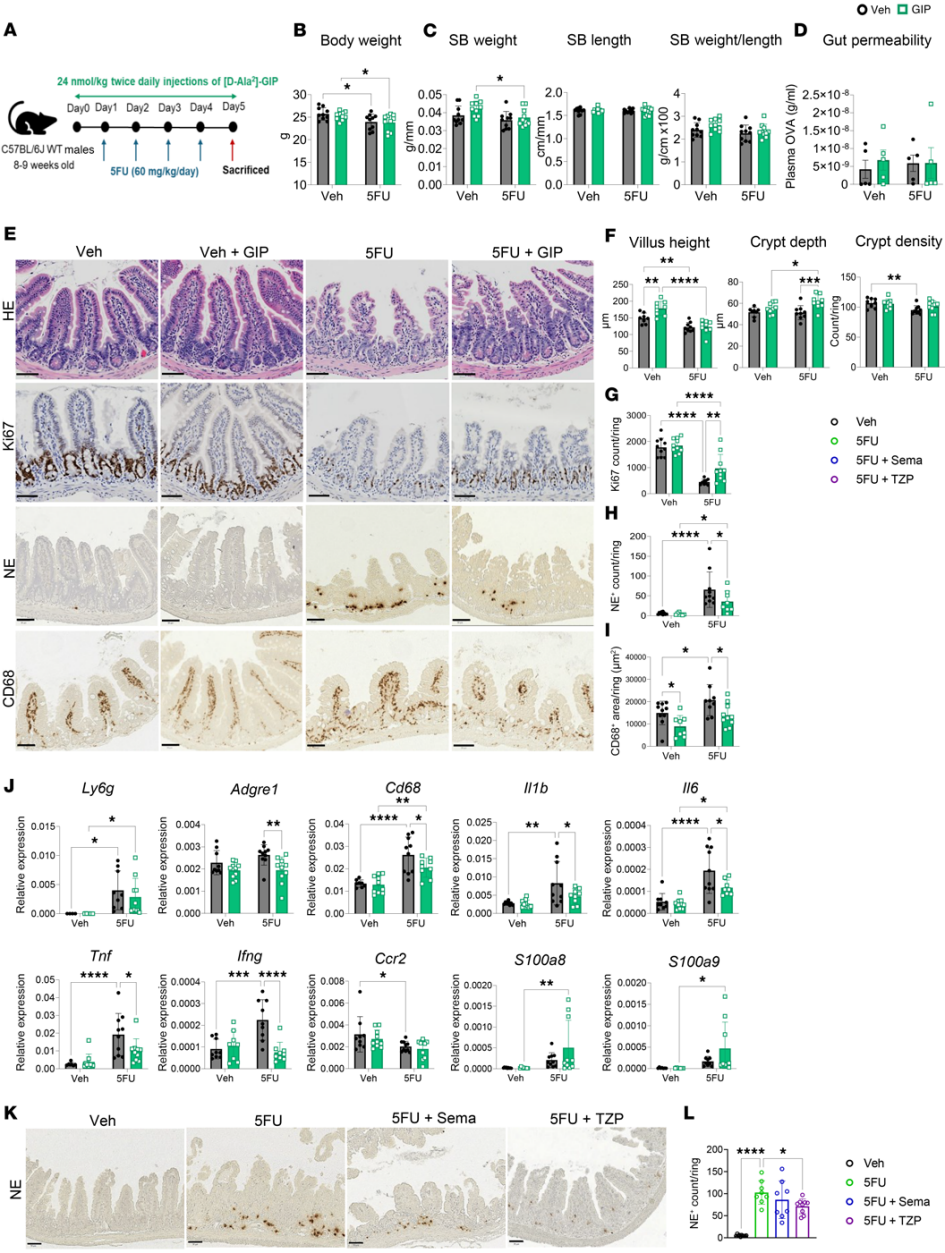
GIPR agonism protects against high-dose 5FU–induced gut damage and inflammation. (**A**) Schematic representation of the experimental protocol. (**B**–**D**) Body weight, small bowel weight and length adjusted for tibia length as well as SB weight/length ratio (*n* = 10), and gut permeability measured as the concentration of plasma ovalbumin 3 hours after oral ovalbumin gavage (*n* = 5). (**E**) Representative images for ileum stained with H&E, anti-Ki67, anti-neutrophil elastase (anti-NE), and anti-CD68 antibody (magnification, 20×). Scale bar: 50 μm. (**F**) Quantification of villus height, crypt depth, and crypt density (*n* = 8–9). (**G**) Average number of Ki67^+^ cells per ring (*n* = 9–10). (**H**) Average number of NE^+^ cells per ring (*n* = 7–10). (**I**) Average positive area of CD68^+^ signal per ring (*n* = 8–10). (**J**) Ileal gene expression relative to Ppia of inflammatory markers in response to 5FU and [DAla2 ]-GIP coadministration (*n* = 9–10). (**K** and **L**) Representative images (magnification, 20×). Scale bar: 50 μm. Quantification of anti-NE staining within the ileum of mice treated with either Veh, 5FU, 5FU and semaglutide (Sema, 10 nmol/kg/day), or 5FU and tirzepatide (TZP, 3 nmol/kg/day) (*n* = 8–10). Data are presented as mean ± SD of samples pooled from 2 independent mouse cohorts. *P ≤ 0.05, **P ≤ 0.01, ***P ≤ 0.001, and ****P ≤ 0.0001 by 2-way ANOVA followed by Tukey post hoc tests (**B**–**D**, **F**–**J**) and by 1-way ANOVA followed by Dunnett’s test with 5FU as the control (**L**).

Given that the selected dose of 5FU, initially chosen to study hematopoiesis (12), resulted in limited intestinal damage and inflammation, the experiment was repeated using more frequent injections of 5FU to enhance the severity of gut injury and inflammation (Figure 2A). Administration of 60 mg/kg/day of 5FU over 4 consecutive days led to body weight loss in both vehicle- and GIP-treated mice (Figure 2B), but it did not perturb SB weight, length, or gut permeability (Figure 2, C and D). However, more frequent 5FU administration induced intestinal injury characterized by blunting of villus height and a reduction in crypt density in the ileum (Figure 2, E and F). GIPR agonism increased villus height and crypt depth in the vehicle-treated and 5FU-treated mice, respectively (Figure 2F). GIPR agonism also attenuated the extent of decreased cellular proliferation in the 5FU-treated mice as assessed by the number of Ki67^+^ cells in the ileum (Figure 2G). GIP treatment also reduced neutrophil activation and the number of macrophages within the ileum in response to 5FU, as evidenced by a reduction in the number of neutrophil elastase^+^ (NE^+^) and CD68^+^ cells, respectively (Figure 2, H and I). Furthermore, GIPR agonism attenuated the 5FU-induced upregulation of several proinflammatory genes within the ileum, including the immune cell markers adhesion G protein-coupled receptor E1 (*Adgre1*) and *Cd68*, and the cytokines *Il1b*, *Il6*, *Tnf*, and *Ifng* (Figure 2J). There was no GIP treatment effect on gene expression of lymphocyte antigen 6 family member G (*Ly6g*), *Ccr2*, S100 calcium binding protein A8 (*S100a8*), or S100 calcium binding protein A9 (*S100a9*) (Figure 2J).

We next used the same protocol to examine the effect of GLP-1R agonism using semaglutide (Sema) and GIPR–GLP-1R coagonism using TZP on 5FU-induced intestinal inflammation (Supplemental Figure 3A). While both treatments led to similar reductions in body weight, SB weight, and the SB weight/length ratio (Supplemental Figure 3, B and C), only TZP significantly attenuated 5FU-induced neutrophil activation within the ileum (Figure 2, K and L). Neither TZP nor Sema treatment modified the effect of 5FU on villus height, crypt depth, or crypt density (Supplemental Figure 3, D and E). Similarly, there was no Sema or TZP treatment effect on the number of mucosal Ki67^+^ and CD68^+^ cells (Supplemental Figure 3, F and G). Expression levels of *Adgre1*, *Cd68*, *Il1b*, *Il6*, *S100a8*, *S100a9*, and *Tnf* showed no change following either treatment (Supplemental Figure 3H). However, the expression level of *Ifng* was downregulated, whereas *Ly6g* was upregulated in the TZP-treated mouse group compared with 5FU treatment alone (Supplemental Figure 3H).

### Gipr^–/–^ mice exhibit increased sensitivity to 5FU-induced gut injury and inflammation in the ileum.

**Figure 3:**
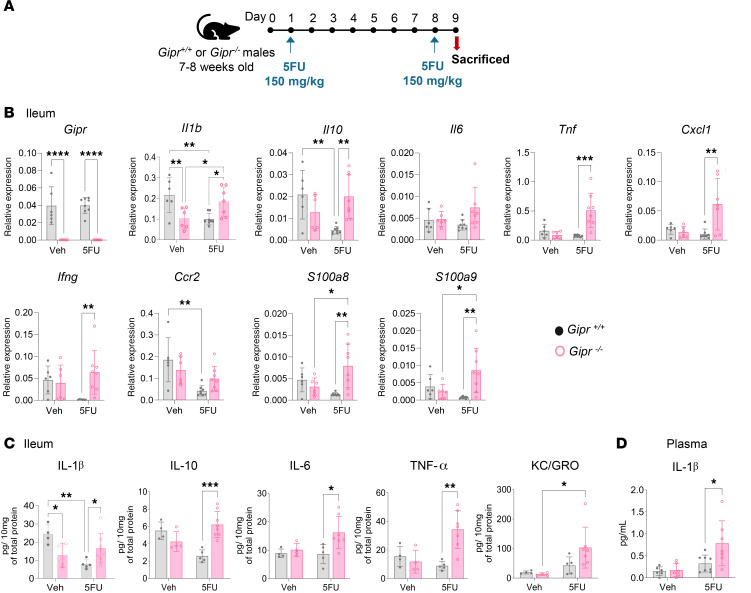
*Gipr^–/–^* mice exhibit increased sensitivity to 5FU-induced gut inflammation. (**A**) Schematic representation of experimental protocol performed. (**B** and **C**) Gene expression relative to *Tbp* (**B**) and protein expression (**C**) of inflammation-related markers within the ileum of *Gipr^+/+^* and *Gipr^–/–^* mice with or without 5FU exposure (*n* = 4–7). (**D**) Circulating IL-1β concentrations (*n* = 6–8). Data are presented as mean ± SD of samples pooled from 3 independent mouse cohorts. *P ≤ 0.05, **P ≤ 0.01, ***P ≤ 0.001, and ****P ≤ 0.0001 by 2-way ANOVA followed by Tukey post hoc tests.

To assess the role of physiological GIPR signaling in the intestinal response to 5FU, we analyzed *Gipr^–/–^* mice. In mice exposed to the intermittent doses of 5FU (Figure 3A), the gene expression levels for *Il1b*, *Il10*, *Tnf*, chemokine ligand 1 (*Cxcl1*), *Ifng*, *S100a8*, and *S100a9* were upregulated in the ileum of 5FU-treated *Gipr^–/–^* mice (Figure 3B). Moreover, ileal protein content of IL-1β, IL-10, IL-6, and TNF-α was increased in 5FU-treated *Gipr^–/–^* mice (Figure 3C). The plasma concentration of the proinflammatory cytokine IL-1β was also elevated in 5FU-treated *Gipr^–/–^* mice (Figure 3D). There was no consistent genotype effect observed on gene expression levels of inflammatory markers within the proximal SB (i.e., the duodenum and jejunum) (Supplemental Figure 4, A and B). Circulating levels of IL-10, KC/GRO, TNF-α, IFN-γ, and IL-6 were not different between groups (Supplemental Figure 4C). Furthermore, mouse body weights and SB biometry were not different between *Gipr^+/+^* and *Gipr^–/–^* with or without 5FU administration; however, spleen weight was reduced in all 5FU-treated groups (Supplemental Figure 5A). Histological analysis of the ileum revealed reductions in crypt depth in response to 5FU, but there was no genotype effect on crypt depth, villus height, or crypt density (Supplemental Figure 5, B and C).

**Figure 4:**
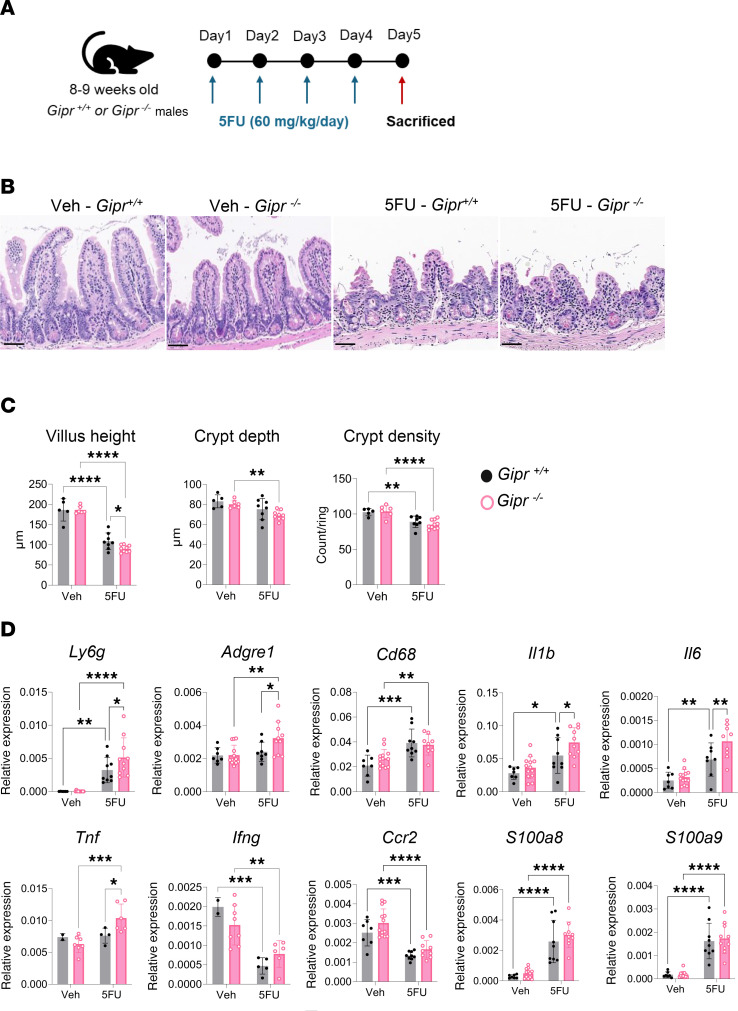
*Gipr^–/–^* mice exhibit increased sensitivity to high-dose 5FU–induced gut damage and inflammation in the ileum. (**A**) Schematic representation of the experimental protocol. (**B**) Representative images for ileum stained with H&E (magnification, 20×). Scale bar: 50 μm. (**C**) Quantification of ileum villus height, crypt depth, and crypt density (*n* = 5–9). (**D**) Gene expression relative to *Ppia* of inflammatory markers within the ileum in response to 5FU in *Gipr^+/+^* or *Gipr^–/–^* mice (*n* = 2–13). Data are presented as mean ± SD of samples pooled from 2 independent mouse cohorts. *P ≤ 0.05, **P ≤ 0.01, ***P ≤ 0.001, and ****P ≤ 0.0001 by 2-way ANOVA followed by Tukey post hoc tests.

Repeated daily exposure to 5FU (Figure 4A) induced significant injury in the mouse ileum characterized by villus blunting as well as a reduction in crypt depth and crypt density (Figure 4, B and C). *Gipr^–/–^* mice exhibited higher sensitivity to 5FU-induced gut injury indicated by a further decrease in villus height and crypt depth (Figure 4, B and C). Both *Gipr^+/+^* and *Gipr^–/–^* mice had lower body weight and SB weight after 5FU exposure (Supplemental Figure 6, A and B); however, *Gipr^–/–^* mice had a higher SB weight/length ratio compared with the 5FU-treated *Gipr^+/+^* mice (Supplemental Figure 6B). *Gipr^–/–^* mice also had upregulated *Ly6g*, *Adgre1*, *Il1b*, *Il6*, and *Tnf* mRNA transcripts in the ileum (Figure 4D). 5FU treatment dysregulated the expression levels of *Cd68*, *Ifng*, *Ccr2*, *S100a8*, and *S100a9*, but there was no discernible genotype effect in response to 5FU (Figure 4D). Similarly, there was no difference in gut permeability, cellular proliferation (Ki67^+^ cell count/ring), neutrophil activation (NE^+^ cell count/ring), and the number of macrophages (CD68^+^ area/ring) in *Gipr*^+/+^ versus *Gipr*^–/–^ mice exposed to high-dose 5FU (Supplemental Figure 6, C–G).

### BM-specific Gipr deletion does not increase 5FU-induced inflammation in the ileum.

Previous studies demonstrated that increased adipose tissue inflammation in *Gipr*^–/–^ mice could be attributed to loss of immunosuppressive GIPR^+^ myeloid cells in the BM that contributed to adipose tissue macrophage populations (11, 12). Accordingly, we assessed whether BM-derived *Gipr*-expressing cells modulate gut inflammation induced by 5FU. BM was transplanted from *Gipr^–/–^* or *Gipr^+/+^* donor mice expressing the CD45.2 allele into irradiated WT recipient mice expressing the CD45.1 allele. The resulting WT^BM-Gipr+/+^ and WT^BM-Gipr–/–^ mice were then treated with 5FU (Supplemental Figure 7A).

**Figure 5:**
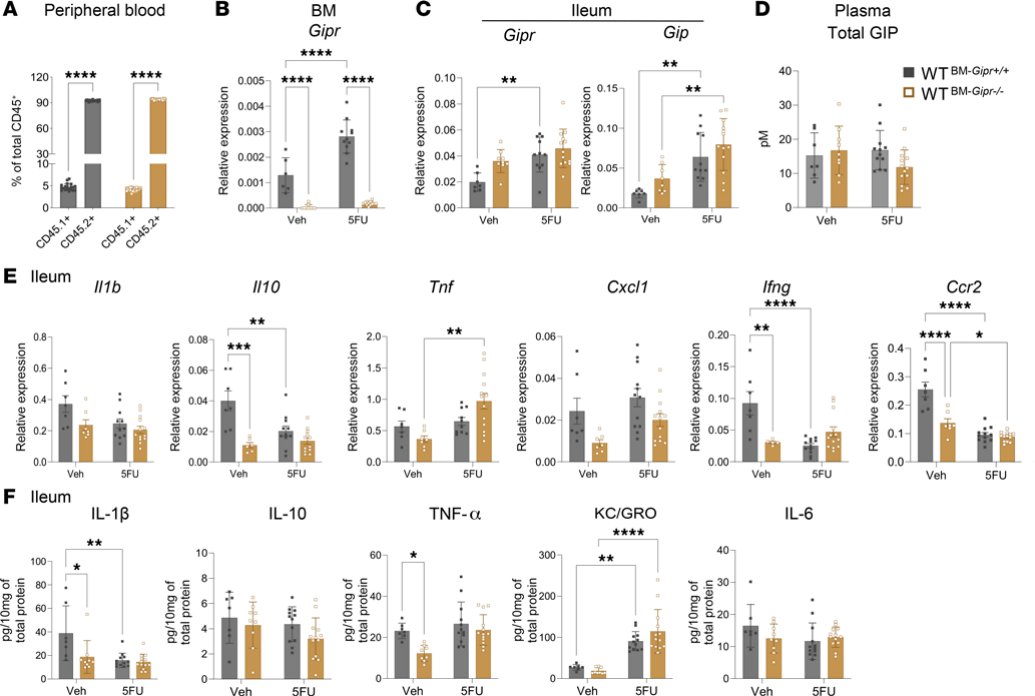
BM-specific *Gipr* deletion does not increase 5FU-induced gut inflammation. (**A**) Percentage of CD45.1^+^ and CD45.2^+^ cells out of total CD45^+^ cells in the peripheral blood of WT CD45.1 recipient mice transplanted with BM from *Gipr*^+/+^ or *Gipr*^–/–^ CD45.2 donor mice (WT^BM-Gipr+/+^ versus WT^BM-Gipr–/–^) (*n* = 20) as depicted in Supplemental Figure 7A. (**B**) *Gipr* mRNA expression relative to *Rpl32* in BM (*n* = 6–12). (**C**) *Gipr* and *Gip* mRNA expression relative to *Tbp* in the ileum (*n* = 7–13). (**D**) Total plasma GIP concentration (*n* = 7–13). (**E** and **F**) Ileal gene expression (**E**) relative to *Tbp* and protein expression (**F**) of inflammation-related markers (*n* = 7–13). Data are presented as mean ± SD of samples pooled from 4 independent mouse cohorts. *P ≤ 0.05, **P ≤ 0.01, ***P ≤ 0.001, ****P ≤ 0.0001 by 2-way ANOVA followed by Tukey post hoc tests.

Efficiency of BM reconstitution in recipient mice was determined by analysis of the percent of CD45.1^+^ and CD45.2^+^ (from total CD45^+^ cells) in peripheral blood, revealing that 90% of the cells were CD45.2^+^ (Figure 5A). Gene expression within the BM showed WT^BM-Gipr–/–^ mice exhibited ablation of *Gipr* expression versus WT^BM-Gipr+/+^ mice (Figure 5B). However, *Gipr* expression within the ileum was not downregulated in response to BM-specific *Gipr* deletion (Figure 5C). Interestingly, 5FU treatment upregulated expression of both BM and ileal *Gipr* in WT^BM-Gipr+/+^ mice (Figure 5, B and C). Similarly, ileal *Gip* expression was upregulated in response to 5FU treatment in both WT^BM-Gipr+/+^ and WT^BM-Gipr–/–^ mice (Figure 5C). Plasma GIP levels were not different between groups (Figure 5D). Tissue biometry and histological analysis of the ileum revealed no genotype effects on spleen weight, SB weight, crypt depth, and density after 5FU treatment (Supplemental Figure 7, B–D). However, villus height was blunted in 5FU-treated WT^BM-Gipr+/+^ but not in WT^BM-Gipr–/–^ mice (Supplemental Figure 7D).

Intriguingly, in the absence of 5FU, WT^BM-Gipr–/–^ mice exhibited lower ileal *Il10*, *Ifng*, and *Ccr2* mRNA transcripts compared with WT^BM-Gipr+/+^ mice (Figure 5E). However, mRNA biomarkers of inflammation, including *Il1b*, *Il10*, *Tnf*, *Cxcl1*, *Ifng*, *Ccr2*, *Il6*, *S100a8*, and *S100a9*, were not dysregulated in the ileum of 5FU-treated WT^BM-Gipr–/–^ compared with 5FU-treated WT^BM-Gipr+/+^ mice (Figure 5E and Supplemental Figure 7E). Similarly, ileal protein expression levels of IL-1β and TNF-α were reduced in vehicle-treated WT^BM-Gipr–/–^ mice compared with vehicle-treated WT^BM-Gipr+/+^ mice, whereas the levels of IL-1β, IL-10, TNF-α, KC/GRO, and IL-6 protein were not different between 5FU-treated groups (Figure 5F). Consistent with the protein cytokine expression within the ileum, circulating concentrations of IL-1β were reduced in the vehicle-treated WT^BM-Gipr–/–^ mice compared with vehicle-treated WT^BM-Gipr+/+^ mice (Supplemental Figure 7F). Plasma concentrations of TNF-α were increased in 5FU-WT^BM-Gipr–/–^ compared with 5FU-treated WT^BM-Gipr+/+^ mice (Supplemental Figure 7F). Circulating KC/GRO was elevated in all 5FU-treated mice independent of genotype, while circulating IL-6 was only elevated in the 5FU-WT^BM-Gipr–/–^ compared with vehicle- WT^BM-Gipr–/–^ mice (Supplemental Figure 7F). IL-10 and IFN-γ plasma concentrations were not different between groups (Supplemental Figure 7F). Therefore, while there are some modest genotype effects on gut and plasma inflammatory markers, knocking out the BM *Gipr* did not completely phenocopy the extent of 5FU-induced gut inflammation observed in *Gipr*^–/–^ mice.

### BM derived from Gipr^+/+^ mice suppresses 5FU-induced gut inflammation in the context of global Gipr deficiency.

**Figure 6:**
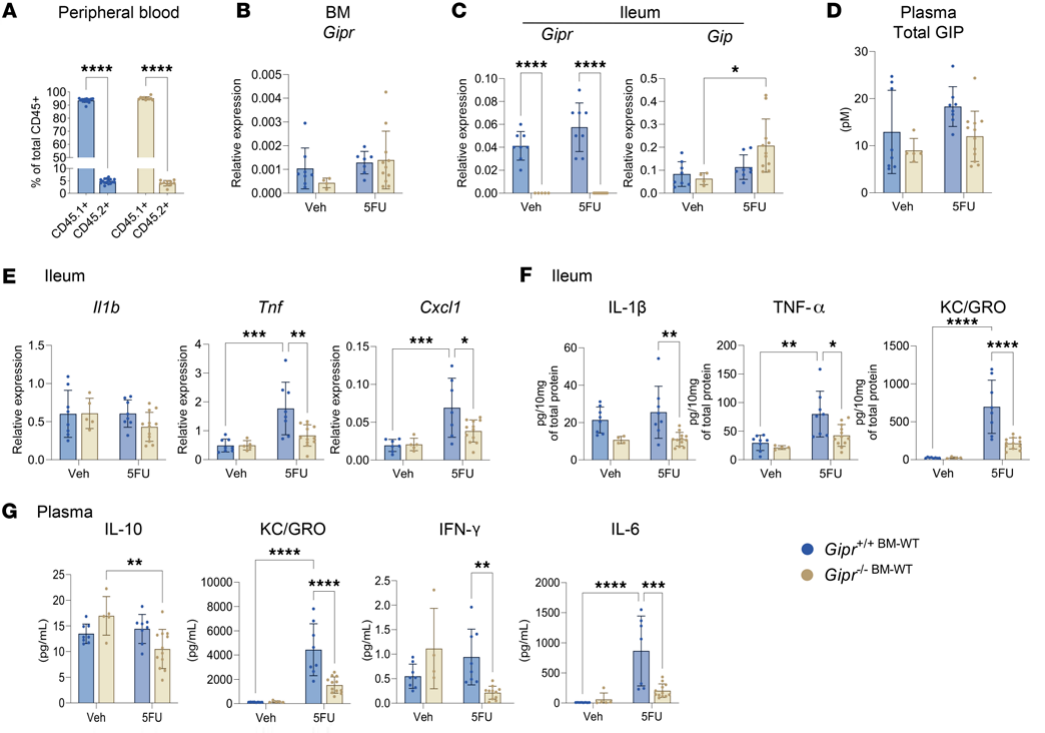
BM-derived *Gipr-*expressing cells suppress 5FU-induced gut inflammation in the context of global *Gipr* deficiency. (**A**) Percentage of sorted CD45.1^+^ and CD45.2^+^ cells out of total CD45^+^ cells in the peripheral blood of *Gipr^–/–^* and *Gipr^+/+^* CD45.2 recipient mice transplanted with BM from WT CD45.1 mice (i.e., *Gipr*^+/+BM-WT^ versus *Gipr*^–/–BM-WT)^ (*n* = 10–16) as depicted in Supplemental Figure 8A. (**B** and **C**) BM *Gipr* expression relative to *Rpl32* (*n* = 4–11) and ileal *Gipr* and *Gip* expression relative to *Tbp* in *Gipr*^+/+BM-WT^ and *Gipr*^–/–BM-WT^ mice with or without 5FU exposure (*n* = 5–12). (**D**) Total plasma GIP concentration (*n* = 5–12). (**E** and **F**) Ileal gene expression (**E**) relative to *Tbp* and protein expression (**F**) of cytokines (*n* = 5–12). (**G**) Plasma cytokine concentrations (*n* = 5–12). Data are presented as mean ± SD of samples pooled from 3 independent mouse cohorts. *P ≤ 0.05, **P ≤ 0.01, ***P ≤ 0.001, ****P ≤ 0.0001 by 2-way ANOVA followed by Tukey post hoc tests.

We next interrogated whether BM-derived *Gipr*-expressing cells modulate the extent of 5FU-induced gut inflammation by transplanting BM from WT CD45.1 donor mice into *Gipr*^–/–^ or *Gipr*^+/+^ CD45.2 recipient mice (Supplemental Figure 8A). After transplantation, mice were designated *Gipr*^+/+BM-WT^ or *Gipr*^–/–BM-WT^, representing mice with or without *Gipr* deletion in all tissues excluding the BM. Ninety percent of the CD 45^+^ cells in the peripheral blood of the recipient mice expressed the CD45.1 allele (Figure 6A). BM *Gipr* expression was restored in *Gipr*^–/–^ recipient mice and was not different from *Gipr^+/+^* mice, indicating successful BM reconstitution (Figure 6B). However, ileal *Gipr* expression remained ablated in the *Gipr*^–/–BM-WT^ versus *Gipr*^+/+BM-WT^ mice, suggesting minimal contribution of BM-derived *Gipr*-expressing cells to local gut *Gipr* expression (Figure 6C). Ileal *Gip* expression was upregulated in response to 5FU exposure in *Gipr*^–/–BM-WT^ mice (Figure 6C). However, plasma GIP levels were not different in response to treatment or genotype (Figure 6D).

Tissue biometry showed elevated SB weight in the *Gipr*^–/–BM-WT^ compared with the *Gipr*^+/+BM-WT^ mice treated with 5FU (Supplemental Figure 8B). Histological analysis showed that 5FU-treated *Gipr*^–/–BM-WT^ mice had modestly higher ileal villus height compared with 5FU-treated *Gipr*^+/+BM-WT^ mice, but no differences were observed in crypt depth or density (Supplemental Figure 8, C and D). Within the ileum, *Gipr*^+/+BM-WT^ mice treated with 5FU exhibited upregulated gene and protein expression of the proinflammatory cytokine *Tnf*/TNF-α and the chemokine *Cxcl1*/KC/GRO compared with the vehicle-treated groups; an effect that was ameliorated in the *Gipr*^–/–BM-WT^ mice treated with 5FU (Figure 6, E and F). Similarly, protein expression, but not gene expression, of IL-1β was decreased in the *Gipr*^–/–BM-WT^ mice compared with *Gipr*^+/+BM-WT^ treated with 5FU (Figure 6, E and F). Furthermore, plasma levels of KC/GRO, IFN-γ, and IL-6 were lower in 5FU-treated *Gipr*^–/–BM-WT^ versus *Gipr*^+/+BM-WT^ mice (Figure 6G). Gene and protein expression of *Il10*/IL-10, *Il6*/IL-6, *Ifng*, *Ccr2*, *S100a8*, and *S100a9* in the ileum were not different between genotypes (Supplemental Figure 8, E and F). Circulating IL-1β, TNF-α, and IL-10 concentrations were not different between 5FU-treated groups (Figure 6G and Supplemental Figure 8G).

Collectively, these findings implicate BM-derived *Gipr*-expressing cells as important modifiers of the extent of gut inflammation. Since WT BM does not influence local *Gipr* expression within the gut of *Gipr*^–/–^ mice, these findings suggest an indirect role for *Gipr*-expressing BM-derived cells in modulating local gut-tissue inflammation.

### Gipr is predominantly localized to nonimmune cells within the lamina propria of the murine SB.

**Figure 7:**
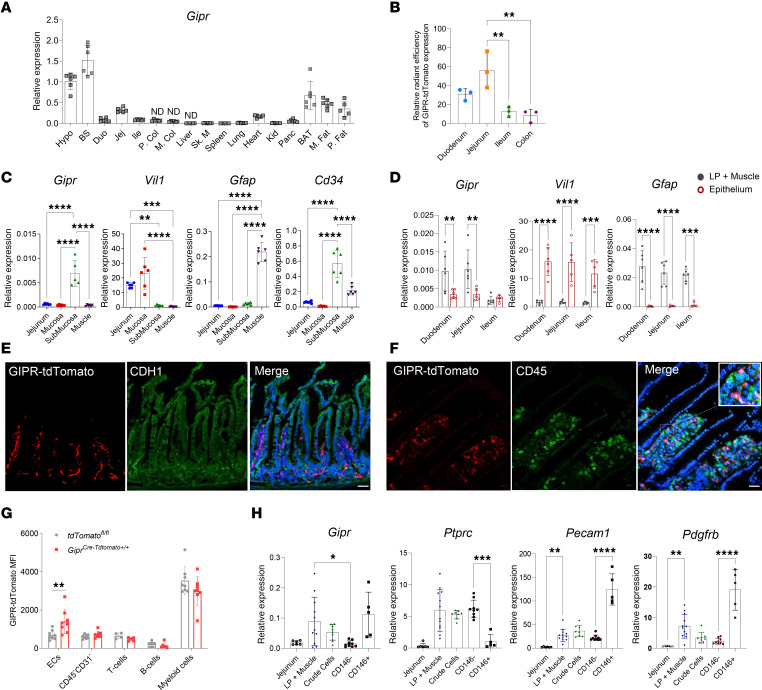
*Gipr* is predominantly expressed in nonimmune cells within the lamina propria of the small bowel. (**A**) Relative *Gipr* expression across various tissues. (**B**) Relative radiant efficiency expression levels of tdTomato from *Gipr^Cre–tdTomato+/+^* mice normalized to the average radiant efficiency expression of *tdTomato^fl/fl^* across gut segments (*n* = 3). (**C**) Gene expression relative to *Rpl32* in manually dissected small bowel compartments (*n* = 6). (**D**) mRNA expression relative to *Rpl32* in lamina propria (LP) + muscle and epithelium throughout distinct segments of the small bowel (*n* = 5–6). (**E** and **F**) Confocal microscopy of jejunum segments showing expression of GIPR-tdTomato (red), DAPI (blue), and E-cadherin (CDH1) (green) (**E**) or CD45 (green) (**F**) (magnification, 40×). Scale bar: 20 mm. (**G**) GIPR-tdTomato mean fluorescence intensity (MFI) of distinct cell populations isolated from the small bowel (*n* = 4–8). (**H**) Gene expression in whole jejunum, LP + muscle, total cells after digestion (crude cells), and isolated CD146^–^ and CD146^+^ cells via magnetic cell separation (*n* = 5–11). Data are presented as mean ± SD from samples from 1 experiment (**A**–**F**) and pooled from 2 independent experiments (**G** and **H**) with each data value corresponding to 1 mouse. *P ≤ 0.05, **P ≤ 0.01, ***P ≤ 0.001, ****P ≤ 0.0001 by 1-way ANOVA followed by Tukey post hoc tests. BAT, brown adipose tissue; BS, brain stem; Duo, duodenum; ECs, endothelial cells; Hypo, hypothalamus; Ile, ileum; Jej, jejunum; Kid, kidney; M. Col, medial colon; M. Fat, mesenteric fat; ND, not detected; P. Col, proximal colon; P. Fat, perirenal fat; Panc, pancreas; Sk. M, skeletal muscle.

To ascertain the relative abundance and potential localization of *Gipr* mRNA transcripts along the gastrointestinal tract, we compared relative *Gipr* mRNA expression in multiple tissues and gut segments. *Gipr* expression was identified in the hypothalamus, brainstem, duodenum, jejunum, ileum, colon, lung, heart, and adipose tissue (Figure 7A). Levels of *Gipr* mRNA transcripts were highest in the hypothalamus and brainstem, followed by adipose tissue (Figure 7A). Within the gut, levels of *Gipr* mRNA transcripts were comparatively low and were highest in the jejunum (Figure 7A). A similar trend was observed using GIPR reporter mice (*Gipr^Cre.tdTomato/+^*). GIPR-tdTomato expression was detected among all gut segments but was highest in the jejunum in comparison with the ileum and colon (Figure 7B). To localize endogenous *Gipr* expression within the gut using complementary approaches, we analyzed different jejunal subcompartments (i.e., mucosa, submucosa, and muscle layers). The epithelial cell marker Villin (*Vil1*) and the glial cell marker glial fibrillary acidic protein (*Gfap*) were not enriched in the submucosal layer, confirming minimal mucosal or muscle layer contamination (Figure 7C). The submucosa was enriched for the stromal cell marker Sialomucin (*Cd34*) (Figure 7C). *Gipr* mRNA expression was enriched in the submucosal layer, which contains the lamina propria and crypts (Figure 7C). We next isolated the epithelial layer from the lamina propria and muscle across gut segments using EDTA dissociation. Adequate epithelial cell separation from the lamina propria was confirmed via analysis of *Vil1,* which was selectively enriched in the epithelial layer, whereas *Gfap* was enriched in the remaining lamina propria and muscle layer within all gut segments (Figure 7D). *Gipr* mRNA transcripts were enriched within the lamina propria and muscle of the proximal (i.e., duodenum and jejunum) but not distal SB (i.e., ileum) (Figure 7D). *Gipr* expression in the lamina propria was further delineated by costaining *Gipr*-tdTomato–expressing cells with the epithelial cell marker E-cadherin (CDH1) and the immune cell marker CD45 showing that the receptor is not localized to either of these cell types (Figure 7, E and F).

Next, submucosa cells were extracted by tissue digestion from all segments of the small intestine of *Gipr^Cre.tdTomato/+^* and littermate control *tdTomato^fl/fl^* mice. Among CD45^+^ immune cells, CD11b^–^CD3^+^ T cells, CD11b^–^MHCII^+^ B cells, and CD11b^+^ myeloid cells were all low for *Gipr*-tdTomato signal (Figure 7G). *Gipr*-tdTomato fluorescence signals were detected in some CD31^+^CD45^–^ ECs, but not among CD45^–^CD31^–^ nonimmune/EC cells (Figure 7G). GIPR has been previously localized to CD146^+^ mesenchymal cells and pericytes in adipose tissue and the CNS (21, 22). Accordingly, we next examined whether *Gipr* mRNA transcripts were higher within intestinal CD146^+^ fractions, enriched for mesenchymal cells, isolated using magnetic cell separation. Notably, SB CD146^+^ populations were enriched for *Gipr* (Figure 7H). These cells also had higher expression of the pericyte marker platelet-derived growth factor receptor β (*Pdgfrb*) and the EC marker platelet EC adhesion molecule (*Pecam1*), and they were relatively depleted for Protein tyrosine phosphatase receptor type C (*Ptprc*), which encodes for CD45 (Figure 7H). These findings reveal that *Gipr* expression within the gut is not enriched within immune cells of the lamina propria; rather, it is predominantly localized to CD146^+^ cells, which include pericytes and ECs (23).

To further refine *Gipr* localization within the gut, we analyzed publicly available single-cell RNA-Seq (scRNA-Seq) data from the mouse ileum (24); however, *Gipr* expression was not detected in this dataset, although pericytes coexpressing *Pdgfrb* and *Mcam,* which encodes for CD146 (23, 25), displayed a very low *Gipr* signal (Supplemental Figure 9). In the human gut cell atlas (26), *GIPR* was detected in epithelial cells, plasma cells, T cells, myeloid cells, and 2 subsets of mesenchymal cells (Supplemental Figure 10). In the mesenchymal cells, a subset of *MCAM*^+^ and *PDGFRB*^+^ pericytes express *GIPR* (Supplemental Figure 10). Coupled with the enrichment of *Gipr* in mouse gut CD146^+^ cells, our data reveal consistent *Gipr*/*GIPR* expression in mouse and human gut pericytes.

## Discussion

Classical metabolic actions of enteroendocrine peptides include the regulation of nutrient intake, pancreatic enzyme secretion, gut motility, energy absorption, and energy disposal (27, 28). The actions of GIP have evolved from a peptide first described as exhibiting modest inhibition of gastric acid secretion to that of an incretin hormone secreted from the proximal gut, potentiating glucose-dependent insulin secretion (1). Subsequently, GIP was shown to improve insulin sensitivity and reduce food intake, actions supporting the development of GIP-based multiagonists for the treatment of people with T2D and obesity (2, 4). GIP also reduces inflammation in adipose tissue (29), whereas loss of the *Gipr* activates a subset of proinflammatory adipose tissue macrophages that impair insulin action (11). Here, we extend the antiinflammatory actions of GIP to the gut. Activation of GIPR signaling attenuates 5FU-induced gut inflammation, whereas loss of the *Gipr* exacerbates the extent of gut inflammation, highlighting the physiological and pharmacological importance of GIP action for the response to gut injury.

Multiple gut peptides, including GLP-1 (30), interact with the immune system to control inflammation (28). Within the hematopoietic and immune system, *Gipr* expression has been identified in circulating myeloid lineage cells and BM myeloid precursors, giving rise to GIPR^+^ adipose tissue macrophages (10–12). Notably, loss of the myeloid *Gipr* impairs type 2 immunity within murine visceral adipose tissue (31). Indeed, loss of the *Gipr* in myeloid cells leads to enhanced adipose tissue inflammation, mediated in part through upregulation of the S100 calcium binding protein S100A8 in adipose tissue (12). Deletion of the *Gipr* also dysregulates hematopoiesis, principally manifested through impaired myelopoiesis (10). The actions of GIP on BM cells are likely mediated in part through regulation of TLR and Notch-related genes important for hematopoiesis. Similarly, levels of several mRNA transcripts encoding inflammation-regulating proteins were increased in the aorta and liver of dyslipidemic *Gipr*^–/–^ mice with experimental atherosclerosis (32). Hence, GIP acts to suppress experimental inflammation in several tissues, in part through BM-derived myeloid GIPRs.

Here we show that gain and loss of GIPR signaling modulates the extent of experimental gut injury in the ileum, consistent with the antiinflammatory actions demonstrated for GLP-1 and GLP-2 in the gut. Activation of GIPR signaling reduces the extent of gut cytokine and chemokine receptor expression in the context of 5FU administration.

A subset of these antiinflammatory actions were also exhibited by the dual GIPR–GLP-1R coagonist TZP, although TZP is a very weak GIPR agonist at the mouse receptor relative to the human GIP receptor, limiting conclusions about the extent of the antiinflammatory action of TZP in mice (33). Although BM-derived *Gipr-*expressing cells suppressed ileal inflammation in the context of global *Gipr* deficiency, analysis of *Gipr* expression in the gut following BM transplantation did not demonstrate reconstitution of *Gipr^+/+^* cells within the *Gipr*^–/–^ intestine. Hence, unlike the mechanisms involving contributions from BM-derived myeloid cells described for GIPR-dependent regulation of adipose tissue inflammation (11, 12), BM-derived GIPR^+^ immune cells are unlikely to directly mediate the antiinflammatory actions of GIP within the gut mucosa. Since the GIPR is important for myeloid cell differentiation (10, 11), it remains possible that BM GIPR^+^ cells attenuate inflammation indirectly by enhancing myeloid cell activity. Nevertheless, we previously demonstrated that the *Gipr* was not required for the hematopoietic response to 5FU administration in mice (12).

This study also suggests a potential role for GIPR signaling within the gut stromal cell compartment in the protection against gut injury; however, the mechanism of action remains to be elucidated. Gut stromal cells — and, more specifically, gut pericytes — are known to play an important role in the maintenance of tissue integrity and homeostasis. Pericytes directly communicate with the vascular system, regulating EC function, promoting angiogenesis, supporting tissue vascularization, maintaining adequate blood flow, and regulating immune cell trafficking (34–36). Pericytes can also assume stem cell properties and support tissue regeneration after injury (34). Hence, pericytes are a reasonable candidate for the direct actions of GIP within the gut.

Given the paucity of currently available validated antisera for detection of the GIPR protein (37, 38), we used cell purification techniques and RNA analyses to localize *Gipr* expression within the lamina propria of the SB. Notably, *Gipr* mRNA was not enriched in gut immune cells (i.e., CD45^+^ cells). These findings suggest that the GIPR-dependent modulation of gut inflammation in mice is not mediated via a direct local GIPR gut-immune axis. Surprisingly, however, our analysis of published scRNA-Seq data showed that, unlike in mice, the *GIPR* is expressed within human gut immune cells, including myeloid and T cells. Species-specific differences in receptor localization were also recently reported for the *Gipr/GIPR* and *Glp1r*/*GLP1R* in murine versus human adipose tissue and heart, respectively (21, 39, 40), further emphasizing the challenges in generalized attribution of mechanisms based on GPCR localization from preclinical studies.

This study has several limitations. While we describe clear phenotypes for both gain and loss of GIPR signaling on gut injury, myeloid cell count and activation, and cytokine expression within the distal SB, an exact mechanism of action linking a population of GIPR^+^ cells to control of gut inflammation remains to be elucidated. While our study using donor *Gipr^+/+^* BM shows a protective effect against 5FU-induced inflammation in *Gipr*^–/–^ mice, the mechanisms underlying these protective phenotypes have not yet been delineated. Another limitation is that only male mice were used in these studies, as lean female mice are more at risk for significant weight loss after 5FU and GLP-1/GIP agonist interventions, which may interfere with the interpretation of the results. Finally, although we were able to detect the *Gipr* in CD146^+^ cells, more precise cell localization, perhaps with purification of gut pericytes and ECs, may help localize key GIPR^+^ cell type within the gut.

In conclusion, GIP attenuates the inflammatory response associated with gut injury in the murine small intestine. Moreover, loss of the *Gipr* exacerbates the extent of intestinal inflammation, a phenotype partially attenuated by BM-derived *Gipr*-expressing cells. These findings establish the importance of a gut GIP/GIPR BM axis in immunoregulation within the SB. GIPR–GLP-1R coagonists such as TZP are now approved for T2D and obesity, retatrutide — the GIPR-biased triple agonist— is in phase 3 clinical trials (41) and the GIPR antagonist-GLP-1RA, AMG-133, is also being studied in phase 2/3 trials (2, 4); therefore, understanding how gain and loss of GIPR signaling in different tissue compartments modifies the response to gut injury may have translational relevance. Intriguingly, TZP therapy has been postulated to exhibit reduced aversive and gastrointestinal side effects in part due to central antiaversive actions of GIP (42); however, a role for antiinflammatory actions of GIP in the gut has not previously been contemplated. The current data may help inform future studies that examine the efficacy of GIP-based therapies in the reduction of clinical adverse effects associated with IBD in patients living with T2D or obesity.

## Methods

### Sex as a biological variable.

Male mice were used in these experiments due to the much greater sensitivity of female mice to 5FU-induced gut injury, resulting in much greater weight loss and illness in the animals. To date, all the actions described for GIP in animals and humans have been ultimately conserved in both males and females.

### Animal models and experiments.

Mice were housed at The Centre for Phenogenomics animal facility at 21°C on a 12-hour light/dark cycle with ad libitum access to water and a standard rodent chow diet (18% kcal from fat, 2018 Harlan Teklad). All GIPR gain-of-function experiments were carried out in male mice on a C57BL/6J background received from The Jackson Laboratory (no. 000664). Animals were given i.p. injections with 24 nmol/kg [DAla^2^]-GIP (Chi Scientific) or vehicle (phosphate-buffered saline [PBS]) twice daily (9 am and 5 pm) for a total of 8 days with 2 i.p. doses of 150 mg/kg 5FU (Mount Sinai Hospital Pharmacy) given at day 1 and day 7. Then, mice were sacrificed on day 8 as previously described for the interrogation of hematopoiesis (12). The same GIPR gain-of-function experiment was repeated with a more severe 5FU protocol utilizing 60 mg/kg/day of 5FU over 4 consecutive days to induce greater gut injury. Mice were treated with either vehicle or 24 nmol/kg [DAla^2^]-GIP twice daily for 5 consecutive days starting 1 day prior to the onset of the 5FU protocol. Similarly, to study the effects of GLP-1R agonism and GLP-1R/GIPR coagonism on the modulation of 5FU-induced gut injury, mice were treated with a once-daily s.c. injection of 10 nmol/kg of Sema (Ozempic, Novo Nordisk), 3 nmol/kg of TZP (Mounjaro, Eli Lilly), or vehicle for 5 consecutive days. Mice were cotreated with 4 daily doses of 60 mg/kg of 5FU (Supplemental Figure 3A). On day 5, 24 hours after the last 5FU injection, all mice were sacrificed for blood and tissue collection. The GIPR loss-of-function studies were similarly performed using both the old and new 5FU protocols in mice with whole-body *Gipr*^−/−^ and WT (*Gipr ^+/+^*) mice that were generated, bred, and validated as previously described (37, 43).

For the localization of *Gipr* in the gut, GIPR reporter mice (*Gipr^Cre.tdTomato/+^*) were generated by crossing *Gipr*^cre/+^ mice obtained from Frank Reimann (44) with B6.Cg-Gt(ROSA)26Sortm9(CAG-tdTomato)Hze/J mice obtained from The Jackson Laboratory (no. 007909), enabling the detection of cells currently expressing *Gipr* or originating from *Gipr* expressing cells.

### BM transplantations.

To study the contribution of hematopoietic or BM-derived GIPR to the immune-regulatory response to 5FU administration, 8-week-old WT B6.SJL-Ptprc^a^ Pepc^b^/BoyJ CD45.1^+^ recipient males obtained from The Jackson Laboratory (no. 002014) were irradiated with 1,100 cGy, split into 2 equal doses separated 4 hours apart. Following this, the tail vein was injected with of 5 × 10^6^ congenic (CD45.2^+^) BM cells from C57BL/6J *Gipr*^–/–^ or *Gipr*^+/+^ donor males, as previously described (12, 45). C57BL/6J CD45.2^+^ *Gipr*^–/–^ or *Gipr*^+/+^ recipient males were irradiated and then transplanted with BM cells harvested from WT B6.SJL-Ptprc^a^ Pepc^b^/BoyJ CD45.1^+^ donor males, following a similar protocol. The degree of reconstitution was analyzed by flow cytometry analysis (Gallios, Beckman Coulter) of tail vein blood ~4 weeks after transplantation using CD45.1-PE-Cy7, CD45.2-APC, and CD45.2-FITC antibodies added to the lymphocyte-myeloid and monocyte-neutrophil panels as previously described (12). At 8–16 weeks after BM transplantation, mice were treated with 2 doses of 5FU (150 mg/kg) a week apart before being sacrificed 24 hours after the second 5FU dose. Mice were fasted for 4–5 hours before they were sacrificed.

### Measurement of intestinal permeability using the ovalbumin (OVA) assay.

To test for gut permeability, mice were daytime fasted (5–6 hours) on the last day (day 4) of 5FU injections then administered an oral gavage of 1 mg of OVA suspended in sterile water. Three hours after oral gavage, 5 μL of tail blood was collected from each mouse using heparin-coated capillary tubes. The blood was treated with 10 μL of PBS containing 0.5% Tween 20 and 50 mmol/L EDTA and was then centrifuged for 5 minutes. Plasma was collected and frozen at –80^°^C for future analysis. To assess the OVA plasma concentration that leaked out of the gut after injury, antibody-conjugated carboxylate modified (CML) beads (Thermo Fisher Scientific) were added to the plasma samples and incubated in a 96-well U-bottom plate overnight at 4°C on a plate shaker to capture the plasma OVA antigen. The OVA-CML complex was later pelleted and detected using a primary rabbit anti-OVA polyclonal antibody (GTX21221; GeneTex, 10 μg/mL), and a secondary phycoerythrin-conjugated-F(ab’)2 fragment donkey anti–rabbit IgG polyclonal antibody (711-116-152; Jackson Immunoresearch Laboratories, 0.5 μg/mL). The beads were then resuspended with FACST buffer (1× PBS [311-425-CL, Wisent], 2% heat-inactivated FBS [090150, Wisent], 2 mmol/L EDTA [B10093-34, Em Science], and 0.05% Tween 20 [P1379, MilliporeSigma]) and quantified by flow cytometry as previously described (46).

### Blood and tissue collection.

Mice were sacrificed by CO_2_ inhalation, blood was collected by cardiac puncture, and tissues were dissected, weighed, and immediately frozen in liquid nitrogen. All blood samples for measuring plasma cytokines and total GIP were collected from tail vein into lithium-coated Microvette tubes (Sarstedt) and mixed with a 10% volume of TED (5000 kIU/mL Trasylol [Bayer], 32 mM EDTA, and 0.01 mM Diprotin A [MilliporeSigma]). Samples were kept on ice, and plasma was collected shortly afterward by centrifugation (12,000*g*) and stored at −80°C.

### Analyte measurements.

Plasma and ileal protein concentrations of TNF-α, IL-10, IL-1β, IL-6, KC/GRO, and IFN-γ were measured using the V-PLEX Proinflammatory Panel 1 Mouse Kit (Meso Scale Discovery, K15048D) as per the manufacturer’s instructions. Ileal protein lysates were extracted by homogenizing tissues in a lysis buffer (50 mM Tris [pH 8], 1 mM EDTA, 10% glycerol, 0.067% Brij 35) supplemented with protease inhibitors (MilliporeSigma) using a TissueLyzer II system (Qiagen). Plasma total GIP was analyzed using an ELISA kit as per the manufacturer instructions (Crystal Chem, 81517).

### Gut biometry and histology.

The gut was dissected and flushed with PBS. Then, the entire SB weight and length were measured. For histology measures, two 2 cm segments of the ileum were collected and fixed in 10% formalin for 24 hours before being transferred to 70% ethanol and stored at 4°C for future processing. Samples were then embedded in paraffin. Paraffin-embedded tissue blocks were sectioned into 4 μm–thick slices and mounted onto charged slides (Assure, Epic Scientific). For gut histology, sections were stained with H&E using standard protocols. Sections were scanned using the Hamamatsu Nanozoomer. Using the QuPath-0.3.2 imaging software, crypt depth was measured as crypt base to tip and villus height was measured as villus base to tip of an average of 10–20 longitudinally, well-orientated crypt/villus units per mouse. Crypt density was measured as the total number of crypts/ring and the average from 2–4 ring sections per mouse was calculated.

### IHC.

Sections were deparaffinized and subjected to heat-induced epitope retrieval using citrate buffer (pH 6.0) in a pressure cooker. After retrieval, the sections were incubated with Peroxidase Block (Bloxall; Vector, SP-6000, lot no. ZJ1129) for 10 minutes, followed by washing in TBS-T. The sections were then treated with 2.5% normal horse serum (ImmPRESS HRP horse anti–rabbit IgG Polymer Kit, Vector, MP-7401, ZL0314) for 20 minutes to block nonspecific binding. Subsequently, the sections were incubated with anti–rabbit monoclonal antibody to Ki67 (Abcam, ab16667, GR3341233-19) at a 1:250 dilution in Antibody Diluent (Agilent, S3022, 1172069) for 1 hour at room temperature. Following TBS-T washes, the sections were incubated with ImmPRESS-HRP horse anti–rabbit IgG Polymer Reagent (ImmPRESS HRP Kit, Vector, MP-7401, ZL0314) for 30 minutes. After another rinse in TBS-T, the sections were treated with ImmPACT DAB Peroxidase (HRP) Substrate (ImmPACT DAB Substrate Kit, Peroxidase, Vector, SK-4105, ZK1018) until chromogen development was complete; they were then washed with distilled water. The sections were counterstained with Mayer’s Hematoxylin (Chaptec, HIY0085-500, C150) for 20 seconds and rinsed under warm running water. Finally, the tissue sections were air dried for 20 minutes and cover slipped using Permount.

To measure neutrophil activation and macrophage number, antigen retrieval was performed by boiling slides in 1× TE buffer (pH 9.0). The ileum sections were stained with either anti-NE antibody (Cell Signaling Technology, E8U3X, rabbit mAb, 90120; 1:400 dilution) or anti-CD68 antibody (Cell Signaling Technology, CD68 [E3O7V], rabbit mAb, 97778; 1:150 dilution), and the signal for all sections was detected using SignalStain Boost IHC Detection Reagent (HRP, rabbit) (Cell Signaling Technology; 8114P) and developed using the ImmPACT DAB Substrate Kit, Peroxidase (HRP) (Vector Laboratories, SK-4105). All sections were counterstained with hematoxylin.

All sections were scanned using the Hamamatsu Nanozoomer. Using the QuPath-0.3.2 imaging software, the number of Ki67^+^ and NE^+^ cells was counted, and the total positive area for CD68 was averaged over 4–6 ring sections per mouse.

### RNA isolation and gene expression analysis.

For the extraction of total RNA, tissue samples were homogenized in TRI Reagent (Molecular Research Center) using a TissueLyser II system (Qiagen). mRNA was then chloroform extracted, precipitated using isopropanol, washed with 75% ethanol, and reconstituted with DEPC-treated water. First-strand cDNA was synthesized from DNase I–treated total RNA using the SuperScript III and random hexamers (Thermo Fisher Scientific). Reverse transcription reactions were performed for 10 minutes at 25°C, 50 minutes at 50°C, and an additional 15 minutes at 70°C. Gene expression levels were quantified by quantitative PCR (qPCR) using a QuantStudio System and TaqMan Gene Expression Master Mix and Assays (Thermo Fisher Scientific) (Supplemental Table 1). Gene expression levels were calculated as 2^–ΔCT^ relative to the housekeeping genes *Tbp*, *Ppia*, or *Rpl32* as indicated.

### Preparation of single-cell suspensions from the small intestine.

Lamina propria cells were isolated as previously described (7) with minor modifications. Briefly, the entire small intestine was cleaned, flushed with HBSS without calcium or magnesium (HBSS^–/–^, 311-512-CL, Wisent), and cut into 0.5 cm pieces. Gut pieces were transferred to a predigestion solution containing 5 mM EDTA, 5 mM DTT (R0861, Thermo Fisher Scientific), and 2% *v/v* FBS in HBSS^–/–^ + 10 mM HEPES (15630-080, Thermo Fisher Scientific) before being shaken at 37.2*g* at 37°C for 20 minutes. The gut tissue pieces were vortexed briefly, and the supernatant was discarded. The EDTA washes were repeated 2 times. A third wash was performed with HBSS^–/–^ + 10 mM HEPES. Tissues were then collected using a 100 μm strainer, minced, and incubated at 37°C for 30 minutes in a digestion solution containing DNase I (200 KU/mL; MilliporeSigma) and Collagenase D (400 Mandl units/mL; Roche) (47) in HBSS with magnesium and calcium + 10 mM HEPES. The tissues were gently sheared with a syringe needle and strained sequentially through 70 and 40 μm strainers, and single cells were resuspended with a MACS buffer for magnetic cell separation (Miltenyi Biotech).

### Flow cytometry.

Cell suspensions of digested lamina propria and muscle from all small intestinal segments were incubated on ice with fluorochrome-conjugated antibodies in a FACS buffer. The following antibodies were used to stain the different cell populations: CD45 APC-Cy7 (clone 30-F11, BD Biosciences), CD11b PE-Cy7 (clone M1/70, BioLegend), CD31 Percp-Cy5.5 (clone 390, BioLegend), CD3 FITC (clone 145-2C11, BioLegend), and MHCII BV 421 (clone M5/114.15.2, BioLegend). Multiparameter flow cytometry analyses were performed using a FACSCanto II machine (BD Biosciences). Flow cytometry analysis was performed using FlowJo software (BD Biosciences).

### Magnetic cell separation.

Magnetic cell separation was performed using CD146 (LSEC) MicroBeads (Miltenyi Biotech, 130-092-007) as per the manufacturer’s instructions. Both the supernatant, containing the CD146^–^ fraction, and the precipitant, containing the CD146^+^ fraction, were collected and stored in TRI Reagent at –80°C for later RNA extraction and gene expression analyses.

### IVIS imaging.

For in vivo imaging system (IVIS) studies, duodenum, jejunum, ileum, and colon were collected and imaged immediately after euthanasia. Regions of interest from the images obtained were identified and quantified as average radiance using Living Image software 4.0. (Spectral Instruments Imaging).

### Confocal microscopy.

Each segment (i.e., duodenum, jejunum, and ileum) of the small intestine was removed, opened longitudinally, and rolled with the mucosa outward to image the entire tissue in one segment as previously described (48). Tissues were then fixed using 4% PFA for 24 hours, dehydrated in 30% sucrose, and subsequently embedded in OCT freezing media. Sections of approximately 18 μm were obtained using a cryostat (Thermo Fisher Scientific) and blocked with a buffer containing 2% BSA for 1 hour. Sections were stained with CD45 monoclonal Ab (Invitrogen, YW62.3, MA1-80090) at dilution 1:100 and secondary antibody goat anti–rat AF647 (Abcam, AB150167) dilution 1:200 or Ecad monoclonal antibody (BD Biosciences, 610182) dilution 1:100 and secondary antibody donkey anti–mouse AF488 (Jackson ImmunoResearch, 715545150) dilution 1:200 and then mounted with fluorescence mounting medium containing DAPI. Images were taken with a ZEISS Confocal Microscope LSM700 (Micro Imaging GmbH, ZEISS). Image processing was performed with ZEN 2011 SP7 software (ZEISS) calculated by subtraction of the background from each slide, and an average was calculated.

### scRNA-Seq analysis.

Published scRNA-Seq data of the mouse ileum (24) and the human gut cell atlas (26) were reanalyzed for the expression of GIPR. For the mouse data, Uniform Manifold Approximation and Projection (UMAP) plots were generated with a standard pipeline and default parameters using Seurat 4.1.0 (49). Scanpy was used to generate the gene expression plots for the human gut cell atlas (50).

### Statistics.

Data are represented as the mean ± SD. Statistical comparisons were made by 1- or 2-way ordinary ANOVA followed by Tukey or Dunnett post hoc tests as indicated in the figure legends using GraphPad Prism version 8 software. Values considered outliers using Grubbs’ test were excluded from analysis. *P* ≤ 0.05 was considered statistically significant.

### Study approvals.

All animal experiments were approved by the Animal Care Committee of the Mount Sinai Hospital and the Animal Care Use Committee of the Sourasky Medical Center.

### Data availability.

Values for all data points in graphs are reported in the Supporting Data Values file and Supplemental Table 2.

## Author contributions

RH, KDK, JAK, LLB, CKW, KEA, and BY designed and executed all mice experiments and tissue analyses. IE, FMG, FR, SF, and CV, conducted the confocal microscopy and flow cytometry experiments on the GIPR reporter mice. DJD designed the experiments and both RH and DJD wrote the manuscript. RH was assigned first in the order of co–first authorship based on relative contribution. All authors reviewed and edited the manuscript prior to submission.

## Supplementary Material

Supplemental data

Supporting data values

## Version 1 12/26/2024

In-Press Preview

## Version 2 02/10/2025

Electronic publication
