# Supplementary material for: Glucose-dependent insulinotropic polypeptide receptor signaling alleviates gut inflammation in mice
Source: JCI Insight. 2024 Dec 26;10(3):e174825. doi: 10.1172/jci.insight.174825 (PMC11948578; doi:10.1172/jci.insight.174825)
Supplement: Supplemental data [file jciinsight-10-174825-s156.pdf]

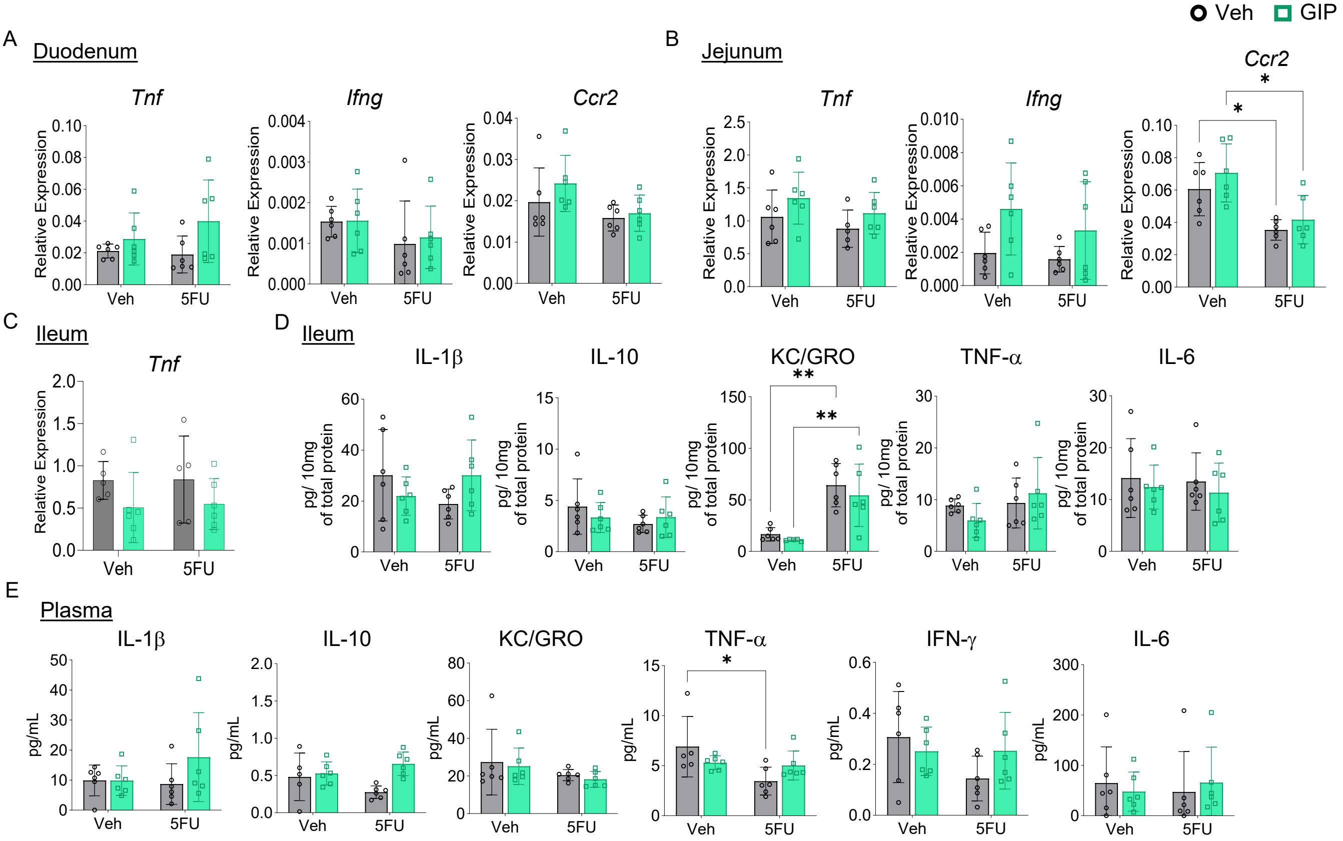

Supplemental Figure 1: Gene and protein expression of inflammation-related markers in the small bowel and circulation of mice co-treated with [DALA²]-GIP and 5FU, related to Figure 1. (A-B) Gene expression, relative to *Tbp*, of inflammation-related markers in the **(A)** duodenum and **(B)** jejunum (n=5-6), and **(C)** Ileum (n=5-6) . **(D)** Ileal protein (n=5-6) and **(E)** plasma concentrations of inflammation-related markers in mice exposed to [D-Ala²]-GIP and 5FU coadministration (n=5-6). Data are presented as the Mean ± SD of samples pooled from three independent mouse cohorts. ∗ P ≤ 0.05, ∗∗ P ≤ 0.01 by two-way ANOVA followed by Tukey post-hoc tests. Abbreviations: 5FU: 5-fluorouracil; *Ccr2*: c-c chemokine receptor-2; GIP: glucose-dependent insulinotropic polypeptide *Ifng*/IFN-γ: interferon gamma; IL-1β: interleukin-1 beta; IL-10: interleukin-10; IL-6: Interleukin-6; KC/GRO: Keratinocyte chemoattractant /human growth-regulated oncogene; *Tbp*: TATA-binding protein; *Tnf*/TNF-α: tumor necrosis factor alpha; Veh: vehicle.

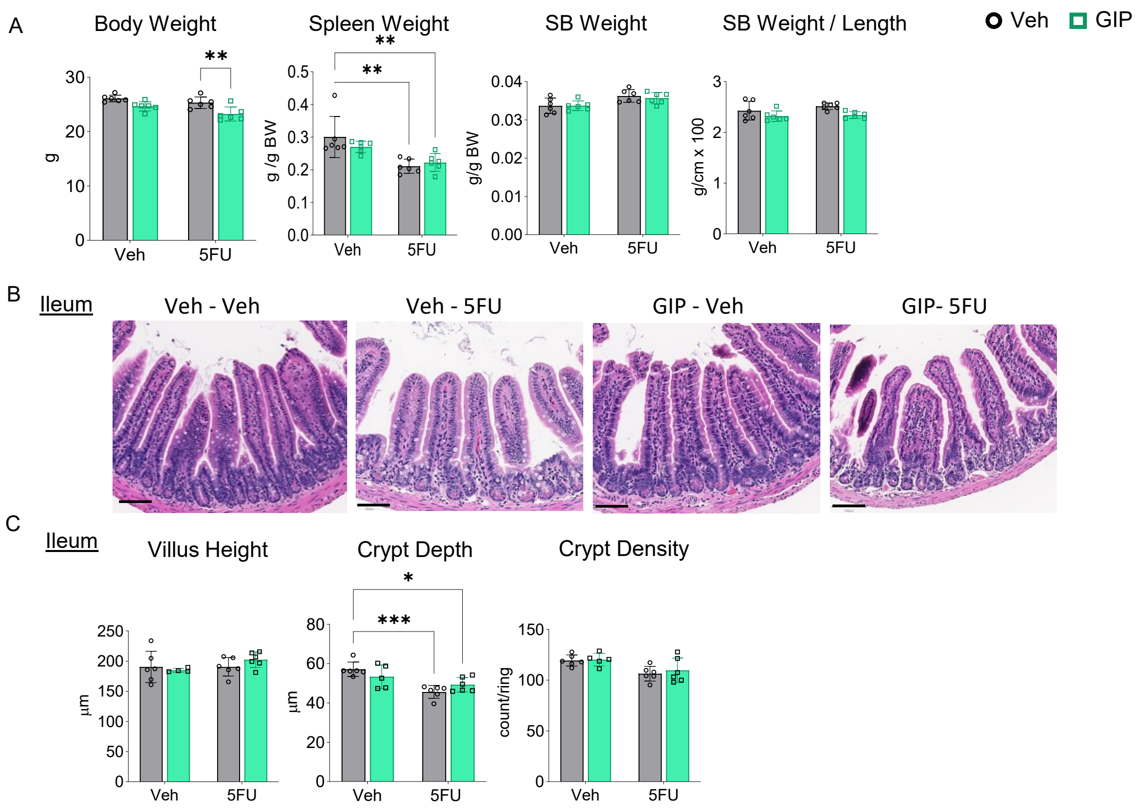

Supplemental Figure 2: Treatment with [D-Ala^2]-GIP twice daily decreases body weight of mice exposed to 5FU, related to Figure 1. (A) Body weight, spleen and small bowel (SB) weights adjusted for total body weight, and SB weight to length ratio of mice exposed to [D-Ala^2]-GIP and 5FU coadministration (n=6). **(B)** Representative histology images of the ileum using H&E staining (20x magnification, scale bar: 100µm). **(C)** Quantification of villus height, crypt depth and crypt density (n=4-6). Data are presented as the Mean ± SD of samples pooled from three independent mouse cohorts. ∗ $P \leq 0.05$, ∗∗ $P \leq 0.01$, ∗∗∗ $P \leq 0.001$ by two-way ANOVA followed by Tukey post-hoc tests. Abbreviations: 5FU: 5-fluorouracil; GIP: glucose-dependent insulinotropic polypeptide; SB: small bowel; Veh: vehicle

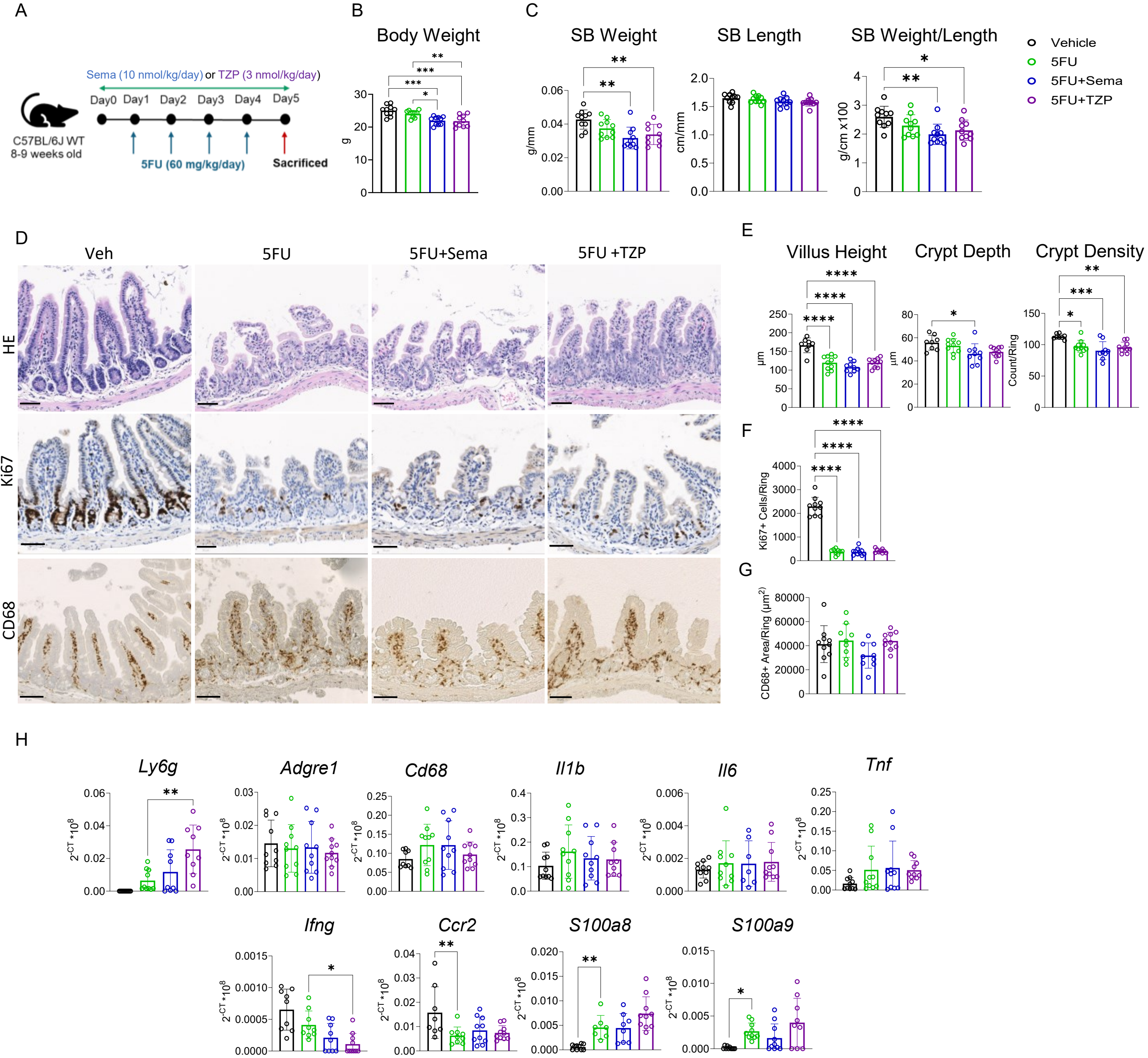

Supplemental Figure 3: Treatment with Semaglutide or Tirzepatide does not modulate 5FU-induced gut injury, related to figure 2. (A) Schematic representation of the experimental protocol. **(B)** Body weight **(C)** Small bowel (SB) weight and length adjusted for tibia length, and SB weight to length ratio (n=10) **(D)** Representative images for ileum stained with hematoxylin and eosin (HE), anti-Ki67, and anti-CD68 antibody (20x magnification, scale bar: 50μm). **(E)** Quantification of villus height, crypt depth and crypt density (n=8-10). **(F)** Average number of Ki67 positive cells per ring (n=9-10). **(G)** Average positive area of CD68+ signal per ring (n=9-10). **(H)** Gene expression of inflammation markers within the ileum of mice treated with Veh, 5FU, 5FU with semaglutide (Sema,10 nmol/kg/day), or 5FU with tirzepatide (TZP, 3 nmol/kg/day) co-treatment (n=9-10). Data are presented as Mean ± SD of samples pooled from two independent mouse cohorts. ∗ $P \le 0.05$, ∗∗ $P \le 0.01$, ∗∗∗ $P \le 0.001$, and ∗∗∗∗ $P \le 0.0001$ by one-way ANOVA followed by Tukey post-hoc tests (B-C) and by Dunnett's test with 5FU as the control (E-H). Abbreviations: 5FU: 5-fluorouracil; *Adgre1*: adhesion G protein-coupled receptor E1; *Ccr2*: c-c-chemokine receptor type 2; *Cd68*: cluster of Differentiation 68; *Ifng*: interferon gamma; *Il1b*: interleukin-1 beta; *Il6*: interleukin-6; *Ly6g*: lymphocyte antigen 6 family member G; *S100a8*: S100 calcium-binding protein A8; *S100a9*: S100 calcium-binding protein A9; SB: Small Bowel; Sema: Semaglutide; *Tnf*: Tumor Necrosis Factor; TZP: Tirzepatide.

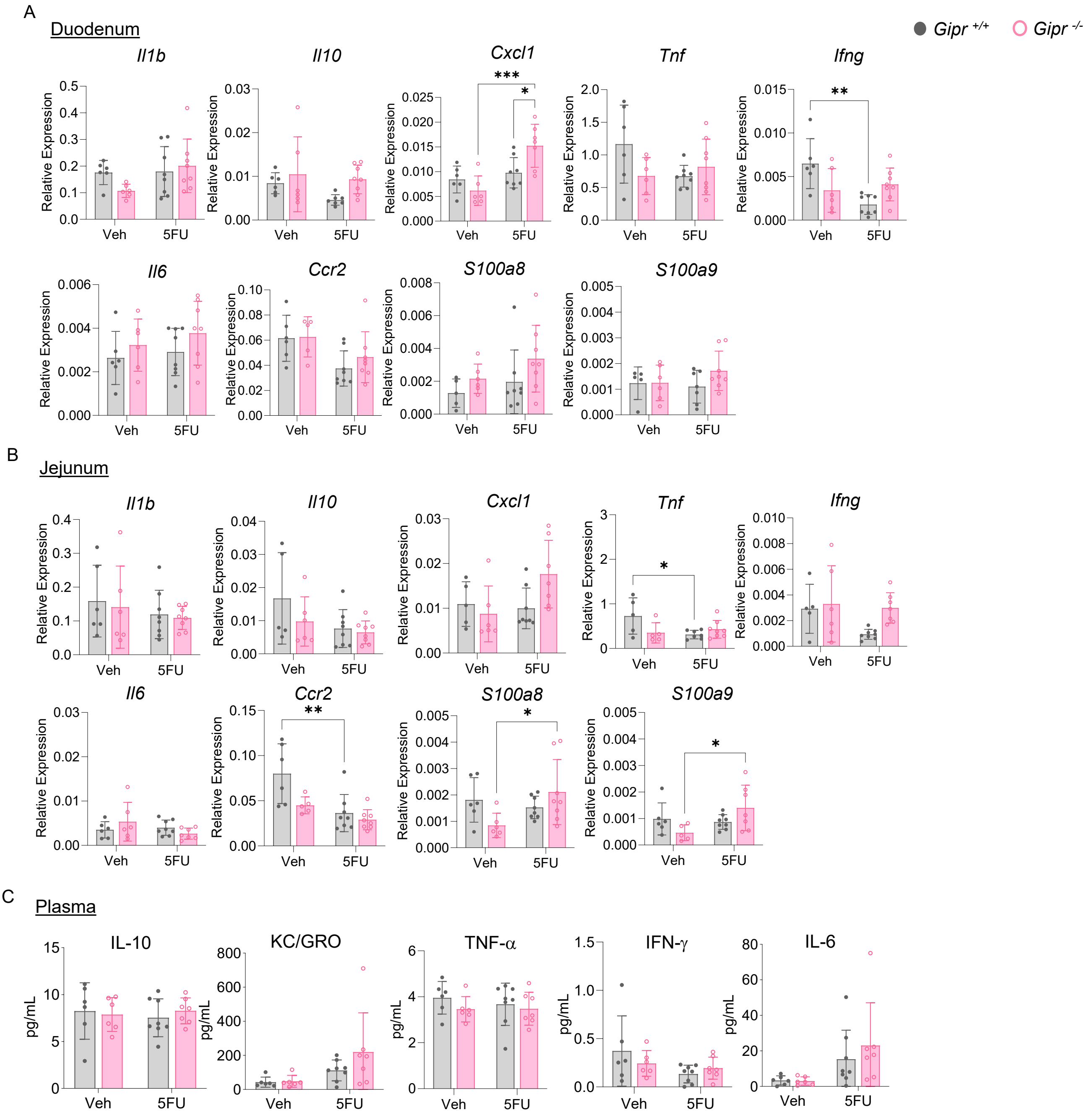

Supplemental Figure 4: Levels of inflammation-related markers in the small bowel and circulation of *Gipr*$^{+/+}$ and *Gipr*$^{-/-}$ mice with or without 5FU exposure, related to figure 3. (A-B) Gene expression, relative to *Tbp*, of inflammation-related markers in the **(A)** duodenum and **(B)** jejunum of of *Gipr*$^{+/+}$ and *Gipr*$^{-/-}$ mice with or without 5FU exposure (n=5-8). **(C)** Circulating cytokine concentrations (n=6-8). Data are presented as Mean ± SD of samples pooled from three independent mouse cohorts. ∗ P ≤ 0.05, ∗∗ P ≤ 0.01, and ∗∗∗ P ≤ 0.001 by two-way ANOVA followed by Tukey post-hoc tests. Abbreviations: 5FU: 5-fluorouracil; *Ccr2*: c-c chemokine receptor-2; *Cxcl1*: chemokine ligand 1; *Gipr*: glucose-dependent insulinotropic polypeptide receptor; *Ifng*/IFN-g: interferon gamma; *Il1b*: interleukin-1 beta *Il10*/IL-10: interleukin-10; *Il6*/IL-6: interleukin-6; KC/GRO: keratinocyte chemoattractant /human growth-regulated oncogene; *S100a8*: s100 calcium-binding protein-8; *S100a9*: s100 calcium-binding protein-9; *Tbp*: TATA-binding protein; *Tnf*/TNF-a: tumor necrosis factor; Veh: vehicle.

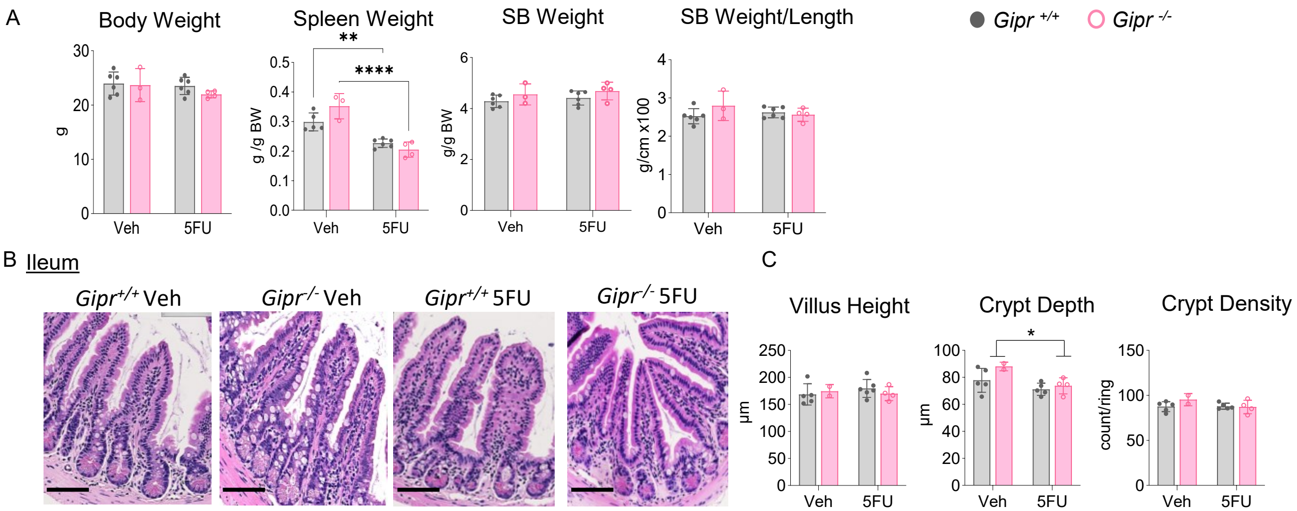

Supplemental Figure 5: Tissue weights and gut morphology in *Gipr*$^{+/+}$ and *Gipr*$^{-/-}$ mice with or without 5FU exposure, related to figure 3 (A) Body weight, spleen and small bowel weights adjusted for total body weight, and small bowel weight to small bowel length ratio of *Gipr*$^{+/+}$ and *Gipr*$^{-/-}$ mice with or without 5FU exposure (n=3-5). **(B)** Representative histology images of the ileum using H&E staining at 20x original magnification (20x magnification, scale bar: 100μm). **(C)** Quantification of villus height, crypt depth and crypt density (n=2-6). Data are presented as the Mean ± SD of samples pooled from three independent mouse cohorts. $*$ $P \leq 0.05$, $**$ $P \leq 0.01$, $****$ $P \leq 0.0001$ by two-way ANOVA followed by Tukey post-hoc tests. Abbreviations: 5FU: 5-fluorouracil; *Gipr*: glucose-dependent insulinotropic polypeptide receptor; SB: small bowel; Veh: vehicle.

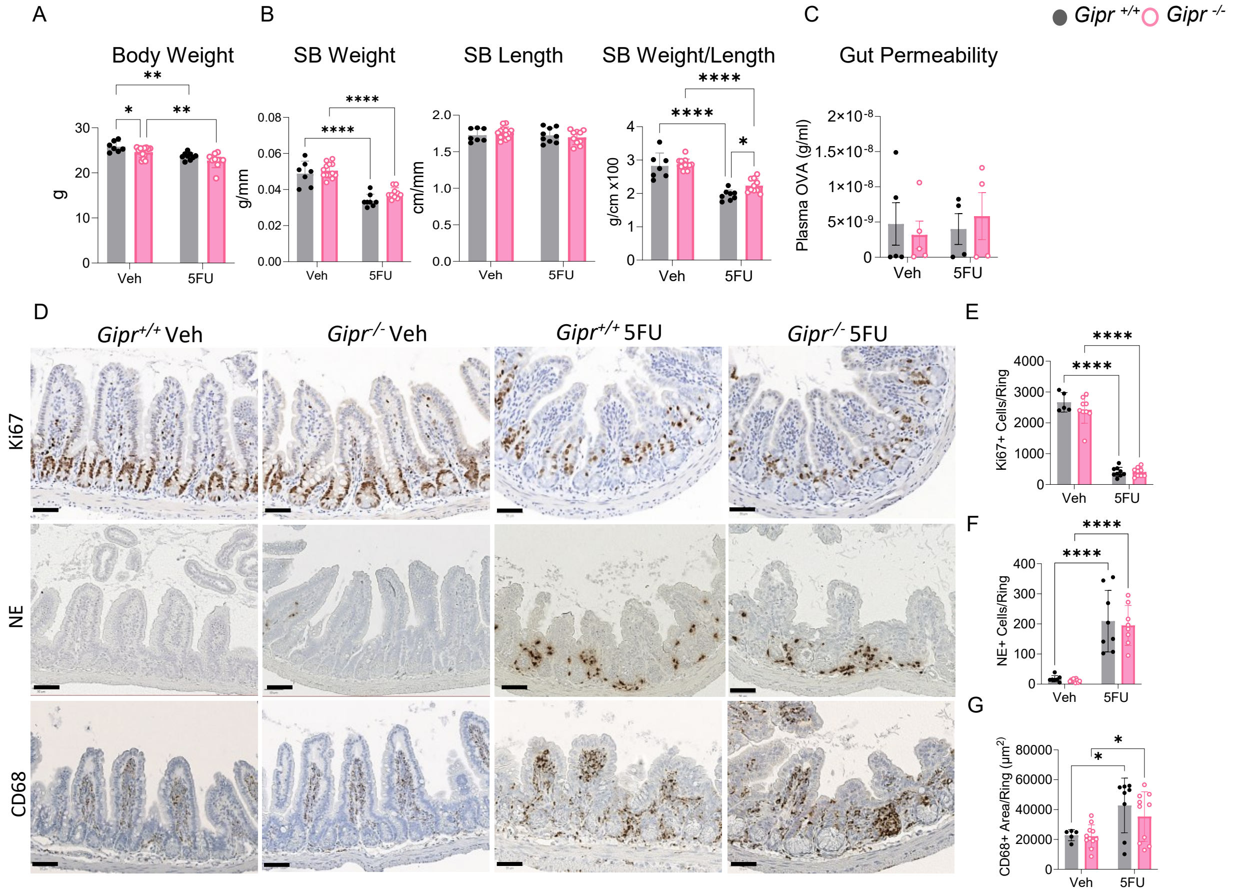

Supplemental Figure 6: Markers of 5FU-induced gut injury in *Gipr-/- and Gipr+/+ mice, related to figure 4.* **(A)** Body weight **(B)** Small bowel (SB) weight and length adjusted to tibia length, and SB weight to length ratio (n=10) **(C)** gut permeability measured as the concentration of plasma ovalbumin 3 hours post oral ovalbumin gavage (n=5). **(D)** Representative images for ileum stained with anti-Ki67, anti-neutrophil elastase (NE), and anti-CD68 antibody (20x magnification, scale bar: 50 µm). **(E)** Average number of Ki67 positive cells per ring. **(F)** Average number of NE positive cells per ring. **(G)** Average positive area of CD68+ signal per ring. Data are presented as Mean ± SD of samples pooled from two independent mouse cohorts. ∗ P ≤ 0.05, ∗∗ P ≤ 0.01, and ∗∗∗∗ P ≤ 0.0001 by two-way ANOVA followed by Tukey post-hoc tests. Abbreviations: 5FU: 5-fluorouracil; CD68: cluster of Differentiation 68; *Gipr*: glucose-dependent insulinotropic polypeptide receptor; NE: Neutrophil Elastase; OVA: Ovalbumin SB: Small Bowel; Veh: vehicle.

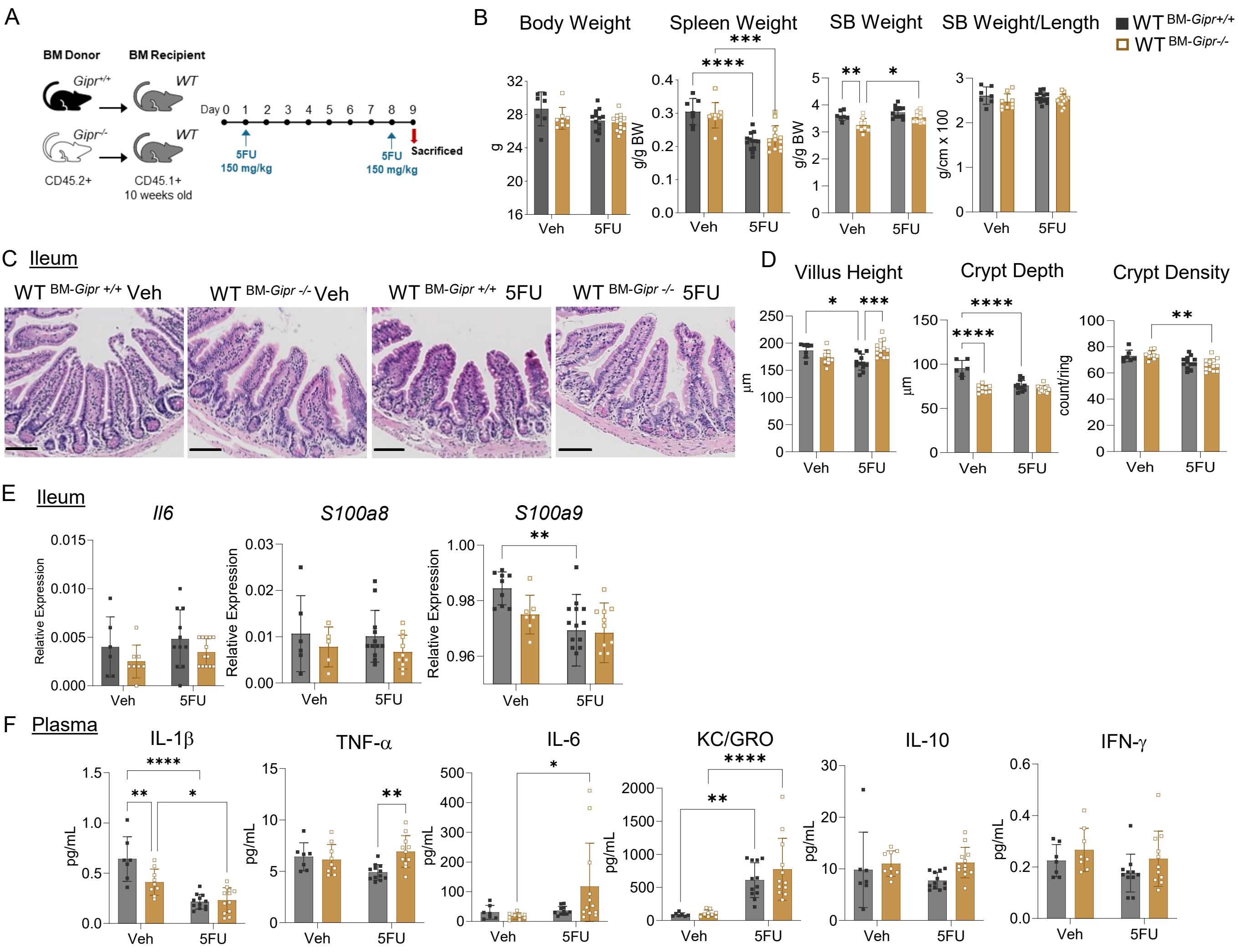

Supplemental Figure 7: Tissue weights, gut biometry and gene and protein expression of inflammation-related markers in WT$^{BM\text{-}Gipr+/+}$ and WT$^{BM\text{-}Gipr-/-}$ mice with or without 5FU exposure, related to figure 5. (A) Schematic representation of the experimental protocol performed in 4 independent experiments. **(B)** Body weight, spleen, and small bowel (SB) weights adjusted for total body weight, and SB weight to length ratio of WT$^{BM\text{-}Gipr+/+}$ and WT$^{BM\text{-}Gipr-/-}$ mice with or without 5FU exposure (n=7-13). **(C)** Representative histology images of the ileum using H&E staining at 20x original magnification (20x magnification, scale bar: 100μm). **(D)** Quantification of villus height, crypt depth and crypt density (n=7-13). **(E)** Ileal gene expression, relative to *Tbp*, of inflammation-related genes (n=6-13). **(F)** Plasma cytokine concentrations (n=7-12). Data are presented as the Mean ± SD of samples pooled from four independent mouse cohorts. * $P \leq 0.05$, ** $P \leq 0.01$, *** $P \leq 0.001$, **** $P \leq 0.0001$ by two-way ANOVA followed by Tukey post-hoc tests. Abbreviations: 5FU: 5-fluorouracil; BM: bone marrow; *Gipr*: glucose-dependent insulinotropic polypeptide receptor; IL-1β: Interleukin-1beta; IL-10: Interleukin-10; *Il6*/IL-6: interleukin-6; KC/GRO: keratinocyte chemoattractant /human growth-regulated oncogene; IFN-γ: interferon gamma; SB: small bowel; *S100a8*: S100 calcium-binding protein-8; *S100a9*: S100 calcium-binding protein-9; TNF-α: tumor necrosis factor alpha; Veh: vehicle.

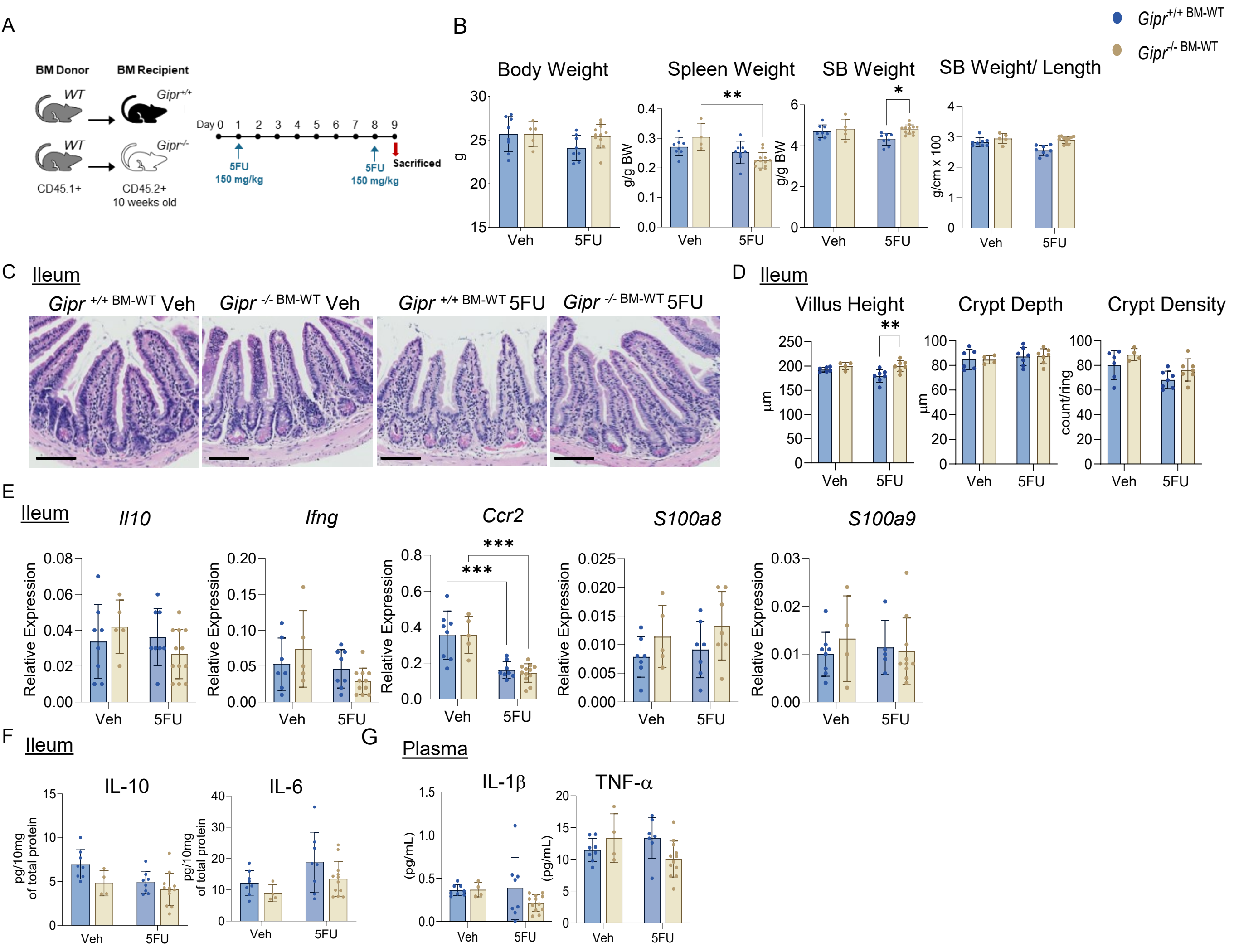

Supplemental Figure 8: Tissue weights, gut biometry and protein and gene expression of inflammation-related markers in *Gipr+/+* BM-WT and *Gipr-/-* BM-WT mice with or without 5FU exposure, related to figure 6. (A) Schematic representation of the experimental protocol performed in 3 independent experiments. **(B)** Body weight, spleen and small bowel (SB) weights adjusted for total body weight, and SB weight to length ratio of *Gipr+/+* BM-WT and *Gipr-/-* BM-WT mice with or without 5FU exposure (n=4-12). **(C)** Representative histology images of the ileum using H&E staining at 20x original magnification (20x magnification, scale bar: 100μm). **(D)** Quantification of villus height, crypt depth and crypt density (n=4-7). **(E)** Ileal gene expression, relative to *Tbp*, of inflammation-related genes (n=5-12). **(F)** Ileal and **(G)** plasma protein cytokine content (n=4-12). Data are presented as the Mean ± SD of samples pooled from three independent mouse cohorts. $*$ $P \leq 0.05$, $**$ $P \leq 0.01$, $***$ $P \leq 0.001$, $****$ $P \leq 0.0001$ by two-way ANOVA followed by Tukey post-hoc tests. Abbreviations: 5FU: 5-fluorouracil; BM: bone marrow; *Ccr2:* c-c chemokine receptor-2; *Gipr*: glucose-dependent insulinotropic polypeptide receptor; IL-1β: interleukin-1 beta; *Il10*/IL-10: interleukin-10; IL-6: interleukin-6; *Ifng*: interferon gamma; SB: small bowel; *S100a8*: s100 calcium-binding protein-8; *S100a9*: s100 calcium-binding protein-9; TNF-α: tumor necrosis factor alpha; Veh: vehicle.

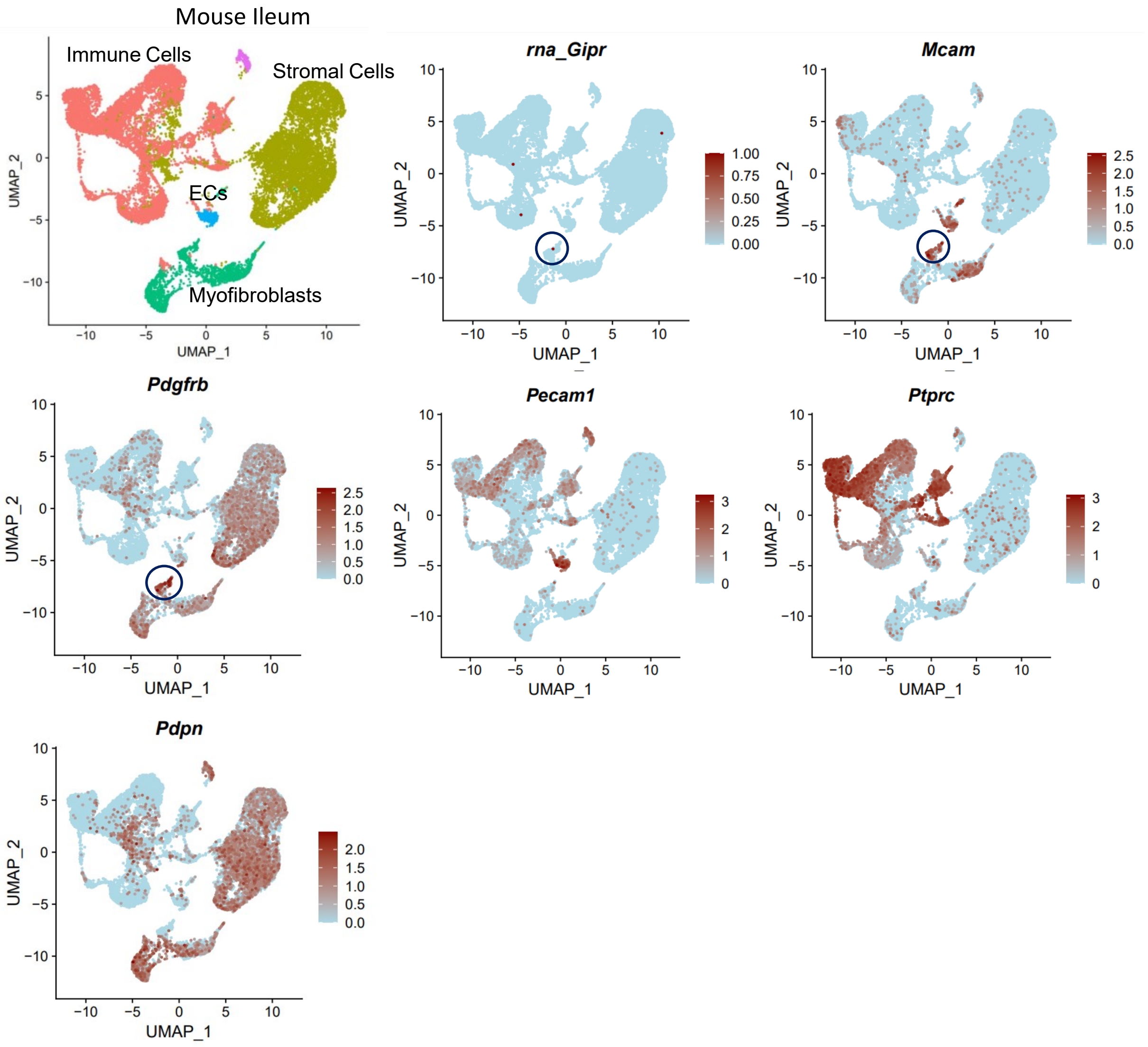

Supplemental Figure 9: Single-cell RNA-seq data on mouse ileal cells showing *Gipr* expression is not detected in the majority of cells, related to figure 7. Markers used to define different cell populations include *Mcam* and *Pdgfrb* for pericytes, *Pdpn* for stromal cells, *Pecam1* for endothelial cells, and *Ptprc* for CD45+ cells. Abbreviations: ECs: endothelial cells; *Mcam:* melanoma cell adhesion molecule; *Pdgfrb:* platelet derived growth factor receptor beta; *Pdpn:* podoplanin; *Pecam1:* platelet/endothelial cell adhesion molecule 1; *Ptprc:* protein tyrosine phosphatase receptor type C.

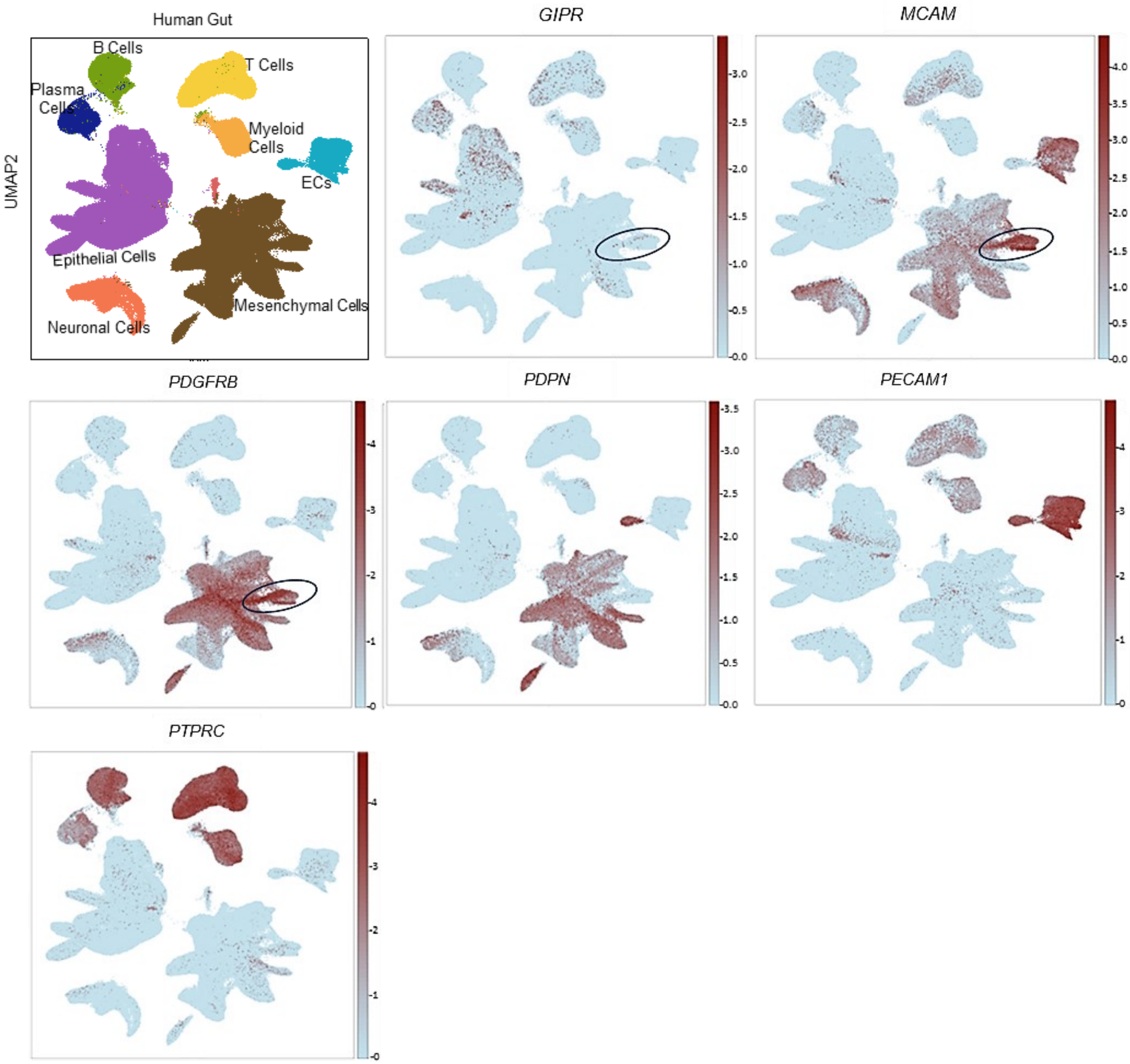

Supplemental Figure 10 : Single-cell RNA-seq data on human gut cells showing *GIPR* expression in immune cells as well as mesenchymal cells, related to figure 7. Markers used to define different cell populations include *MCAM* and *PDGRFB* for pericytes, *PDPN* for stromal mesenchymal cells, *PECAM1* for endothelial cells, and *PTPRC* for CD45+ cells. Abbreviations: ECs: endothelial cells; *GIPR*: glucose-dependent insulinotropic polypeptide receptor; MCAM: melanoma cell adhesion molecule; *PDGFRB:* Platelet-derived growth factor receptor beta; *PECAM1*: Platelet endothelial cell adhesion molecule-1; *PTPRC*: Protein Tyrosine Phosphatase Receptor Type C.

Supplemental Table 1: List of primers used in gene expression analysis

Gene Symbol	Gene Name	Catalog Number
Ccr2	Chemokine (C-C motif) receptor 2	Mm01216173_m1
Cxcl1	Chemokine (C-X-C motif) ligand 1	Mm04207460_m1
Gip	Gastric inhibitory polypeptide	Mm00433601_m1
Gipr	Gastric inhibitory polypeptide receptor	Mm01316349_g1
Ifng	Interferon gamma	Mm01168134_m1
Il10	Interleukin 10	Mm01288386_m1
Il1b	Interleukin 1 beta	Mm01336189_m1
Il6	Interleukin 6	Mm00446190_m1
S100a8	S100 calcium binding protein A8	Mm00496696_g1
S100a9	S100 calcium binding protein A9	Mm00656925_m1
Tnf	Tumor necrosis factor alpha	Mm00443258_m1
Ly6g	Lymphocyte antigen 6 complex, locus G	Mm04934123_m1
Adgre1	Adhesion G protein-coupled receptor E1	Mm00802529_m1
Cd68	Cluster of differentiation 68	Mm03047343_m1
Tbp	TATA box binding protein	Mm00446973_m1
Ppia	Cyclophilin	Mm02342430_g1
Rpl32	Ribosomal protein L32	Mm02528467_g1
